# Identification of Auxin Metabolites in *Brassicaceae* by Ultra-Performance Liquid Chromatography Coupled with High-Resolution Mass Spectrometry

**DOI:** 10.3390/molecules24142615

**Published:** 2019-07-18

**Authors:** Panagiota-Kyriaki Revelou, Maroula G. Kokotou, Violetta Constantinou-Kokotou

**Affiliations:** Chemical Laboratories, Department of Food Science and Human Nutrition, Agricultural University of Athens, Iera odos 75, 11855 Athens, Greece

**Keywords:** UPLC-QToF-MS, *Brassica oleracea*, *Raphanus raphanistrum*, *Eruca sativa*, *Brassica rapa*, *Brassicaceae*, auxin, amino acid conjugates

## Abstract

Auxins are signaling molecules involved in multiple stages of plant growth and development. The levels of the most important auxin, indole-3-acetic acid (IAA), are regulated by the formation of amide and ester conjugates with amino acids and sugars. In this work, IAA and IAA amide conjugates with amino acids bearing a free carboxylic group or a methyl ester group, along with some selected IAA metabolites, were studied in positive and negative electrospray ionization (ESI) modes, utilizing high-resolution mass spectrometry (HRMS) as a tool for their structural analysis. HRMS/MS spectra revealed the fragmentation patterns that enable us to identify IAA metabolites in plant extracts from eight vegetables of the *Brassicaceae* family using a fast and reliable ultra-performance liquid chromatography quadrupole time-of-flight mass spectrometry (UPLC-QToF-MS) method. The accurate *m*/*z* (mass to charge) ratio and abundance of the molecular and fragment ions of the studied compounds in plant extracts matched those obtained from commercially available or synthesized compounds and confirmed the presence of IAA metabolites.

## 1. Introduction

**Figure 1:**
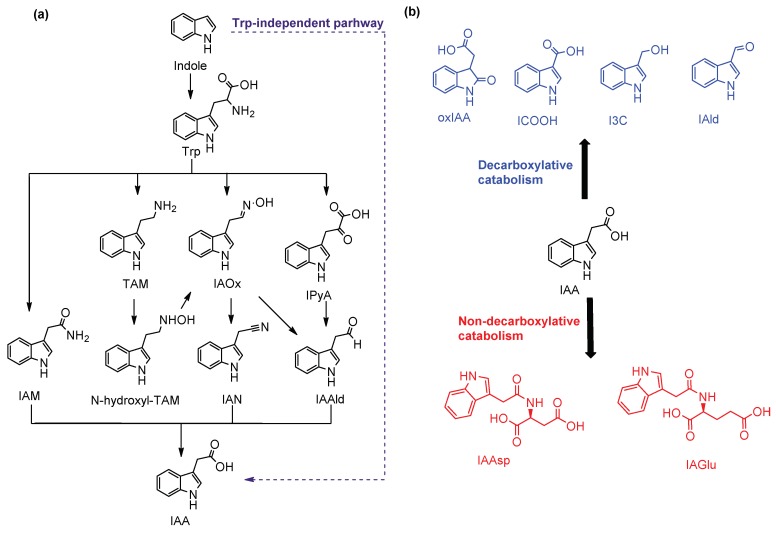
(**a**) Indole-3-acetic acid (IAA) biosynthetic pathways and (**b**) IAA catabolism.

Phytohormones are natural products that regulate all physiological and developmental processes occurring in plants. Auxins were the first class of endogenous plant hormones discovered by Charles and Francis Darwin [1]. The most important auxin, indole-3-acetic acid (IAA), is critically involved in different stages during plant growth, development, and metabolic homeostasis [2], although 4-chloroindole-3-acetic acid (4-Cl-IAA), a naturally occurring chlorinated derivative of IAA, also displays notable auxin activity [3]. IAA biosynthesis occurs via two major routes—tryptophan (Trp)-independent and Trp-dependent pathways. Trp-dependent auxin biosynthesis pathways include the indole-3-pyruvic acid (IPyA) pathway, the tryptamine (TAM) pathway, the indole-3-acetaldoxime (IAOx) pathway, and the indole-3-acetamide (IAM) pathway [4,5] (Figure 1a). Biosynthesis of IAA through the IAOx pathway has been observed primarily in *Cruciferae* [6]. 

The metabolism of IAA occurs through two processes—the decarboxylative and the non-decarboxylative catabolism (Figure 1b). Decarboxylation of IAA is a minor catabolic pathway, catalyzed by plant peroxidases. Major degradation products that generate from IAA catabolism are indole-3-aldehyde (IAld), 2-oxindole-3-acetic acid (oxIAA), indole-3-carbinol (I3C), and indole-3-carboxylic acid (ICOOH) [5]. Catabolism of IAA in *Arabidopsis* is usually performed through a non-decarboxylative process, involving oxidation steps of the indole ring of the free IAA or the IAA conjugates with glucose or aspartic acid (Asp) and glutamic acid (Glu) [5,7]. 

Plants produce various IAA-conjugated forms either with sugars (ester conjugates), or with amino acids, peptides, and proteins (amide conjugates), which contribute to many crucial processes within the plants, such as storage and transport of IAA [7,8], IAA catabolism [9], regulation of IAA levels [10], prevention against enzymatic degradation of IAA [11], and part of abiotic and biotic stress of plants [12,13]. 

Several amide conjugates with the amino acids leucine (Leu), alanine (Ala), Asp, and Glu have been identified in *Arabidopsis* [14,15], while amide conjugates with isoleucine (Ile), valine (Val), phenylalanine (Phe), Asp, Glu, and Ala have been quantified in rice [16]. Auxin conjugates with Asp, Glu, and tryptophan (Trp) have been identified in young tomato leaves, Ginkgo male flower, and coconut milk [17]. Pěnčík et al. [18] isolated IAA amino acid conjugates with Ala, Asp, Glu, Val, Leu, Phe, and glycine (Gly) from *Helleborus niger* by immunoaffinity extraction and quantified them using high-performance liquid chromatography tandem mass spectrometry (HPLC-MS/MS). The sample required pretreatment with diazomethane, prior to analysis, for the transformation of IAA conjugates into their corresponding methyl esters. This analytical protocol, although laborious, does not examine the possibility of the pre-existence of IAA amino acid methyl ester conjugates in the sample. Van Meulebroek et al. [19] identified IAA conjugates with aspartic acid dimethyl ester and glutamic acid dimethyl ester in tomato fruit, along with IAA conjugates of Ala, Asp, Trp, and Val using ultra-high performance liquid chromatography high-resolution Orbitrap mass spectrometry (UHPLC-HR-Orbitrap-MS). In the same study, IAA conjugates with alanine methyl ester, glutamic acid dimethyl ester, isoleucine, Ala, and Asp were identified in tomato leaf [19]. As indicated in the literature, the MS study of IAA conjugates was performed in positive electrospray ionization (ESI) mode [16,17,18,19].

Considering that almost 90% of the total IAA in *Arabidopsis* are amide-linked conjugates with amino acids and peptides [5,20], a need has emerged for more extensive studies focused on the identification of auxin amide conjugates, which will contribute substantially to our knowledge and understanding of the overall mechanism of IAA. Despite the research on IAA conjugates in *Arabidopsis*, no reports yet exist in the literature for studies about other members of the *Brassicaceae* family. 

Identification and quantification of auxins and IAA conjugates is challenging, due to their low concentration (nmol g^−1^ to pmol g^−1^ fresh weight), interferences from other co-extracted substances, and differences in concentration levels between IAA and auxin conjugates [5]. High-resolution mass spectrometric (HRMS) techniques, such as quadrupole time-of-flight (QToF), provide high selectivity against matrix interferences, as well as accurate mass and structural information, allowing the screening, identification, and target analysis of compounds [21]. These techniques have also been previously applied in the elemental composition analysis of fragmentation ions [22,23,24,25] and in the identification of bioactive substances in plant extracts [26,27,28,29,30]. Ultra-high-performance liquid chromatography (UHPLC) allows physical separation of complex elutes. The speed, resolution, and sensitivity of UHPLC make it ideally suited for use with HRMS. The combination of HRMS with UHPLC provides a powerful technique with very high sensitivity [31].

Herein, we report the mass spectrometry study and the fragmentation pathways for 20 IAA amide conjugates with amino acids bearing a free carboxylic group or a methyl ester group, as well as IAA and selected IAA metabolites (IAld, IAN, IAM, and 4-Cl-IAA), using high-resolution mass spectrometry in positive and negative ESI modes. The HRMS study and accurate masses enabled us to elucidate the major fragmentation pathways through the determination of elemental composition analyses of the fragment ions, and to develop a fast and reliable UPLC-QToF-MS method for the identification of the target class of compounds in plant extracts. The application of the method in methanolic extracts of the *Brassicaceae* family vegetables is also presented and a wide range of indoles is identified, which may permit further elucidation of *Brassicaceae* signaling pathways.

## 2. Results and Discussion

### 2.1. High-Resolution Mass Spectrometry Study

MS spectra for all compounds were recorded both in ESI positive and negative ion modes and are given in the Supplementary Materials file along with the extracted ion chromatograms. Characteristic ions that can be used for the identification of IAA metabolites are summarized in Table 1. For the proposed chemical formulae, Agilent MassHunter software was used, taking into account the nitrogen rule, the mass error being lower than 5 ppm, and the isotopic abundance distribution match.

**Figure 2:**
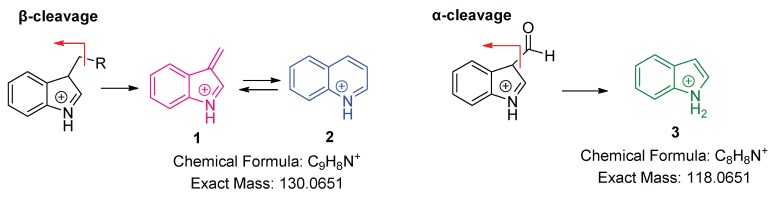
Fragments of 3-substituted indole compounds in the positive ion mode.

In positive ionization mode, indole compounds bearing an alkyl chain as the substituent in the pyrrole ring undergo a cleavage of the β-bond with respect to the aromatic system and lead to 3-methylene-3H-indol-1-ium, **1** (Figure 2). This ion can be rearranged to the more stable quinolinium cation, **2**, with a calculated *m*/*z* 130.0651 [32,33]. MS^2^ spectra of IAA and all studied IAA metabolites, generated from the protonated ions [M + H]^+^, showed the adoption of the same fragmentation pathway. 4-Chloro-indole acetic acid exhibited two chlorinated quinolinium cations with *m*/*z* 164.0252 and *m*/*z* 166.0231, respectively, in a ratio of 3:1, due to the presence of the two chlorine isotopes. Indole-3-carbaldehyde, lacking a β-bond, generated 1*H*-indol-1-ium, **3**, with *m*/*z* 118.0651, due to the cleavage of the α-bond with respect to the pyrrole ring. Prinsen et al. [34] studied IAA and related metabolites in positive ESI mode and suggested the quinolinium cation as a diagnostic ion for IAN, IAM, and IAld determination. The same cation, confirmed by the findings of the current HRMS study, has also been reported as the main fragment of amino acid conjugates IATrp [17], IAAla, IALeu, IAAsp, and IAGlu, [14], generated by the complete breakage of the molecules under the ESI conditions used.

**Figure 3:**
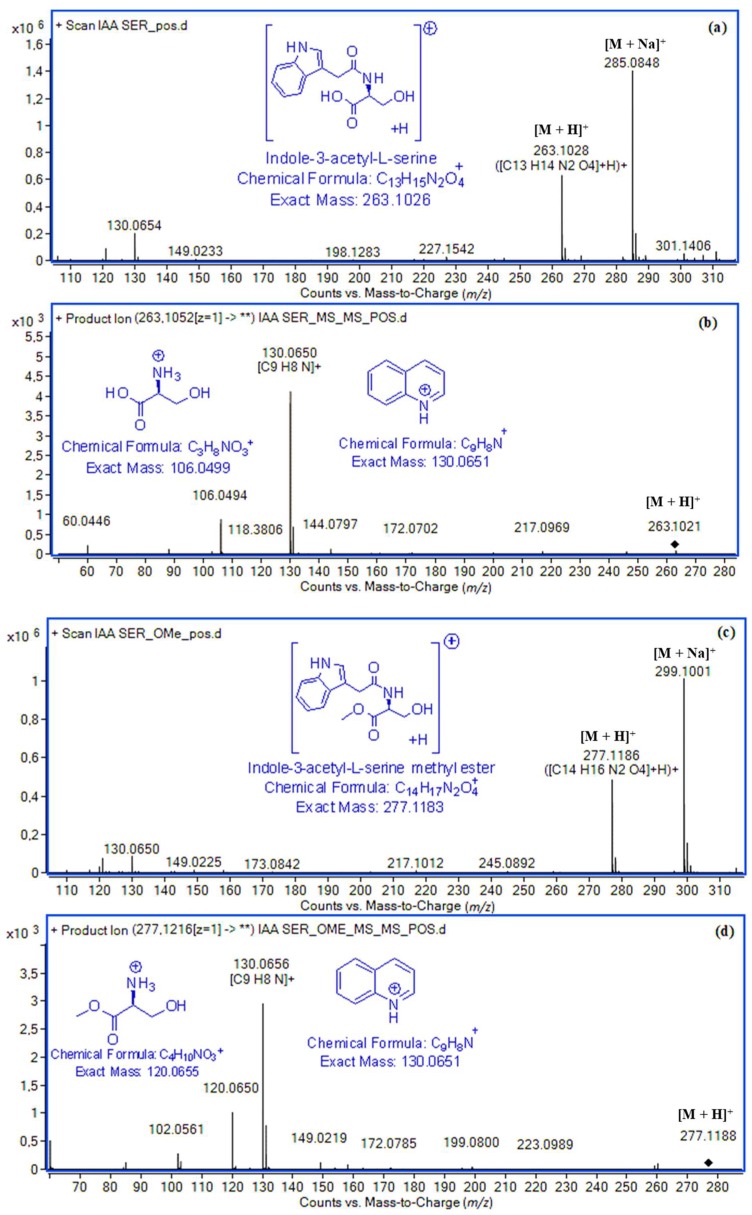
(**a**) Full scan and (**b**) MS/MS spectra of indole-3-acetyl-l-serine in positive electrospray ionization (ESI) mode. (**c**) Full scan and (**d**) MS/MS spectra of indole-3-acetyl-l-serine methyl ester in positive ESI mode.

The full scan spectra of all studied indole amide conjugates which have the amino acid either as a free carboxylic group or as a methyl ester presented the ions [M + H]^+^ and [M + Na]^+^. Their MS^2^ spectra generated from the protonated ions [M + H]^+^ exhibited, apart from the quinolinium cation (exact mass: 130.0651), a second characteristic fragment assigned to the amino acid residue cation, in low to moderate abundances, due to the amide bond cleavage. The quinolinium cation alone is not a strong confirmation of the presence of IAA amino acid conjugates, because it can be generated from a variety of indole compounds that can undergo β-cleavage [32,33,34]. Nevertheless, since every amino acid has a unique nominal molecular mass, the fragment assigned to the amino acid along with the ions [M + H]^+^, [M + Na]^+^, and quinolinium may allow the identification of every IAA amino acid conjugate. As an example, the full scan and MS/MS spectra of indole-3-acetyl-l-serine and indole-3-acetyl-l-serine methyl ester in positive ESI mode are given in Figure 3. Molecular ion [M + H]^+^ with *m*/*z* 263.1028 (exact mass: 263.1026, Δ 0.8 ppm) and fragment ion *m*/*z* 106.0494 (exact mass for protonated serine ion: 106.0499, Δ 4.7 ppm) are related to the presence of IAA amide conjugate with serine in a free carboxylic form. Molecular ion with *m*/*z* 277.1186 (exact mass: 277.1183, Δ 1.1 ppm) and fragment ion with *m*/*z* 120.0650 (exact mass for protonated serine methyl ester ion: 120.0655, Δ 4,2 ppm) are related to the presence of IAA conjugate with serine in the methyl ester form. 

**Figure 4:**
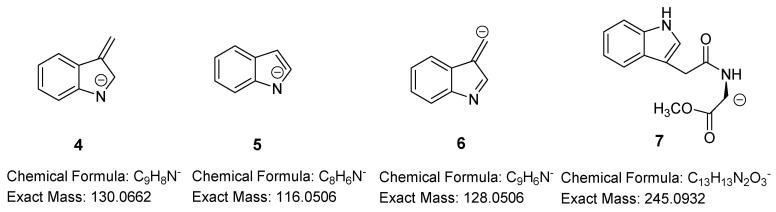
Fragments of 3-substituted indole compounds in the negative ion mode.

In the negative ion mode, IAA and all IAA metabolites exhibited the deprotonated molecular ions [M − H]^−^, which were selected as the precursor ions for the MS^2^ spectra. IAA and IAM gave a fragment with a proposed chemical formula of C_9_H_8_N^−^ (exact mass: 130.0662) corresponding to 3-methyleneindolin-1-ide, **4**, formed after a β-cleavage of the aliphatic chain at C-3 position of the indole (Figure 4). 4-Chloro-indole acetic acid due to the presence of the two chlorine isotopes exhibited two chlorinated fragments with *m*/*z* 164.0271 (exact mass: 164.0273, Δ 1.2 ppm) and *m*/*z* 166.0246 (exact mass: 166.0244, Δ 1.2 ppm), respectively, in a ratio of 3:1. It has been noticed that the quinolinium ion **2** with exact mass *m*/*z* 130.0651 or the 4-chlorinated one with *m*/*z* 164.0252 obtained in positive ion mode and 3-methyleneindolin-1-ide **4** with *m*/*z* 130.0662 or 4-chloromethyleneindolin-1-ide with *m*/*z* 164.0271 obtained in negative ion mode seem to be identical. However, they are different due to their opposite charge and their accurate masses are separated by two electrons. This was also observed in fragmentation pathways of nonsteroidal anti-inflammatory drugs [35]. 

**Figure 5:**
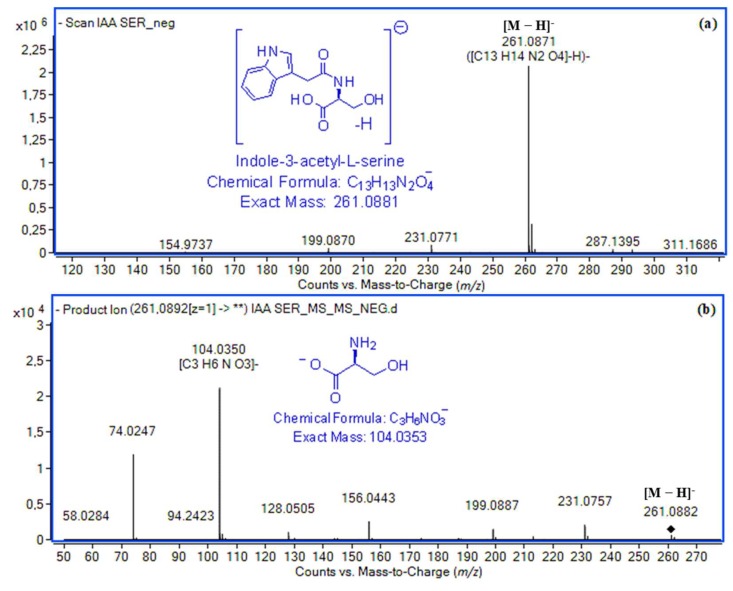

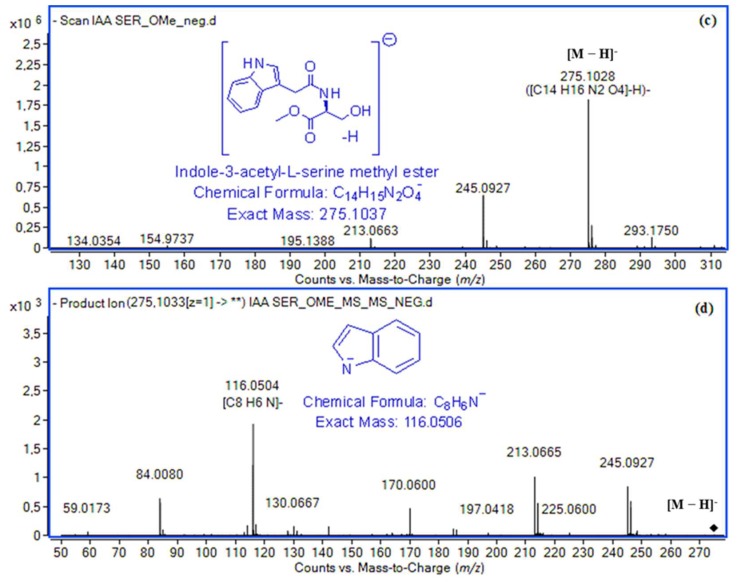
(**a**) Full scan and (**b**) MS/MS spectra of indole-3-acetyl-l-serine in negative ESI mode. (**c**) Full scan and (**d**) MS/MS spectra of indole-3-acetyl-l-serine methyl ester in negative ESI mode.

To the best of our knowledge, there are no existing literature reports on the negative ESI mass spectrometry study of auxin amino acid conjugates. In the MS^2^ spectra of the amide conjugates carrying the amino acid with a free carboxylic group, a prominent fragment ion with a chemical formula depending on the amino acid was observed. These specific fragments were found to correspond to the anion form of the corresponding amino acid, formed as a result of the amide bond cleavage. Both the deprotonated and the fragment ions can be used for the identification and confirmation of the amino acid conjugates with IAA, since each amino acid has a different nominal molecular mass. As an example, in negative ESI mode, indole-3-acetyl-l-serine can be identified by the ion [M − H]^−^ at *m*/*z* 261.0871 (exact mass: 261.0881, Δ 3.8 ppm) of the full scan spectrum (Figure 5a) and the most intense signal of the MS^2^ spectrum at *m*/*z* 104.0350 (Figure 5b). The fragment ion at *m*/*z* 104.0350 has a chemical formula generated using Agilent MassHunter software of C_3_H_6_NO_3_^−^ (exact mass: 104.0353, Δ 2.9 ppm), corresponding to the deprotonated [M − H]^−^ ion of the amino acid L-serine generated after the cleavage of the amide bond.

Amide conjugates with amino acid methyl esters exhibited the deprotonated molecular ions [M − H]^−^, but they seem to follow a different fragmentation pathway compared to that of conjugates with amino acids bearing a free carboxylic group. For the most intense signal found in the MS^2^ spectra of the amide methyl ester conjugates, as well as in the spectrum of 3-IAA-carbaldehyde, the chemical formula of C_8_H_6_N^−^ (exact mass: 116.0506, Δ 0.8 ppm) is proposed and may be attributed to indole-1-ide, **5** (Figure 4), generated after the α-cleavage of the aliphatic chain at C-3 position of the indole. 

IAN exhibited the ion **6** with *m*/*z* 128.0506 due to the loss of a molecule of HCN.

In indole-3-acetyl-l-serine methyl ester MS and MS^2^ spectra (Figure 5c,d), along with the ions at *m*/*z* 275.1028 and 116.0504 matched to the ions [M − H]^−^ and **5**, an ion of *m*/*z* 245.0927 was obtained corresponding to the C_13_H_13_N_2_O_3_^−^ formula. The same ion **7** was also observed for IAA conjugate with Trp, Tyr, and Glu(OMe)_2_ and it is obtained as a result of the amino acid side chain elimination. 

From the present mass spectrometry study, it can be concluded that auxin IAA amide conjugates generated diagnostic amino acid-specific fragment ions, both in positive and negative ion modes, which permit their identification. However, especially for IAA conjugates, positive ion mode seems to be more helpful, although both modes showed almost the same level of reliability and accuracy. In positive ion mode, all IAA conjugates studied, apart from the protonated molecular ion and adduct with sodium, additional diagnostic fragments, fragment **2** related to the presence of the indole ring, and an amino acid-specific fragment, were recorded. Thus, both the indole ring and the amino acid are detected. In negative ion mode, apart from the deprotonated molecular ions, conjugates with an amino acid bearing a free carboxylic group generated the amino acid-specific fragment ion, while conjugates with an amino acid bearing a methyl ester group generated the diagnostic for the indole ring fragment **5**.

### 2.2. Analysis of Brassicaceae Vegetables

The *Brassicaceae* family utilizes the indole ring to produce a diverse set of compounds such as glucosinolates (glucobrassicin), phytoalexins (camalexin), and the phytohormone IAA. The active amount of IAA is tightly regulated by metabolic processes and IAA amino acid conjugates are classified, up to now, as storage and catabolism molecules. 

Since the study of IAA amide conjugates in the *Brassicaceae* family is limited in *Arabidopsis* species and within our project dedicated to the analysis of phytonutrients in cruciferous vegetables [32,36,37,38], we decided to apply our high-resolution mass spectrometry study of the IAA metabolites to explore the presence of IAA amide conjugates in other members of the *Brassicaceae* family. We studied extracts from eight vegetables, namely, *Brassica oleracea* L. var. *botrytis* L. cv. Zarka (white cauliflower), *Brassica oleracea* L. var. *rubra* L. (red cabbage), *Brassica oleracea* L. var. *capitata* L. (white cabbage), *Brassica oleracea* L. var. *italica* Plenck cv. Calabrese (green broccoli), *Brassica oleracea* L. var. *italica* Plenck cv. Viοlleto (purple broccoli), *Raphanus raphanistrum* L. subsp. *sativus* (L.) Domin (radish), *Eruca sativa* (L.) Mill. (arugula), and *Brassica rapa* L. subsp. *rapifera* Metzg. (turnip). The extraction from cruciferous vegetables was performed using a simple extraction method with 80% MeOH, followed by filtration. The simultaneous identification of IAA, IAA metabolites, and amide conjugates using UPLC-QToF-MS was conducted in negative and positive ESI mode. Identification was based on the retention times relative to those of the standard compounds, the accurate mass of ions [M − H]^−^, [M + H]^+^, and the selected fragments from their HRMS/MS spectra which are given in Table 1.

**Figure 6:**
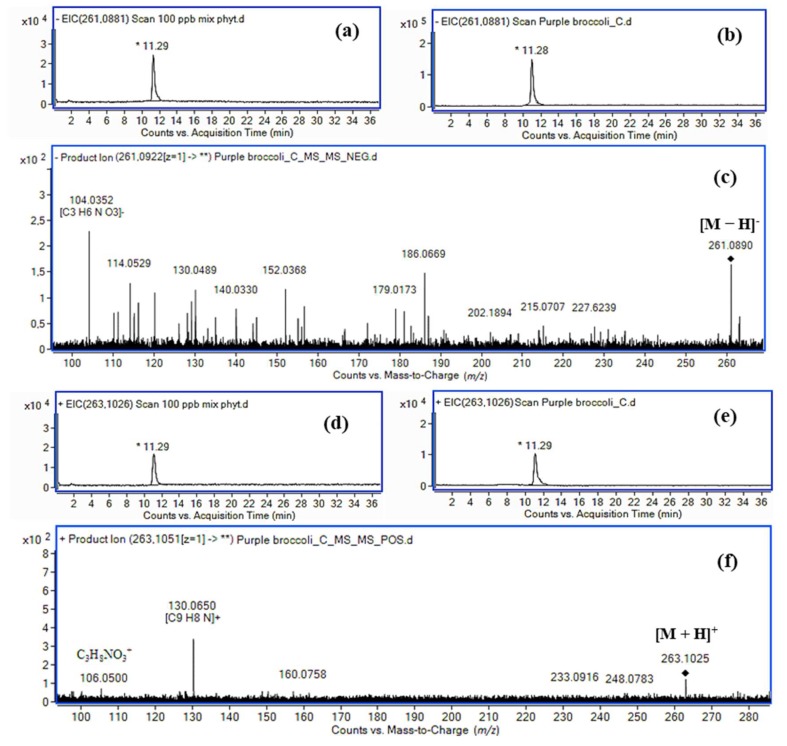
Extracted ion chromatograms for (**a**) a standard solution of IASer, (**b**) a purple broccoli extract, and (**c**) an MS/MS spectrum of the extract in negative ionization mode. Extracted ion chromatograms for (**d**) a standard solution of IASer, (**e**) a purple broccoli extract, and (**f**) an MS/MS spectrum of the extract in positive ionization mode.

Extracted ion chromatograms for a standard solution of IASer and for a purple broccoli extract are presented in Figure 6, along with the MS/MS spectra of the extract in negative and positive ionization modes. The ion at *m*/*z* 261.0890 corresponding to the deprotonated molecular ion of IAA conjugate with serine (exact mass: 261.0881, Δ 3.4 ppm) and the diagnostic fragment ion at *m*/*z* 104.0352 (exact mass: 104.0353, Δ 0.9 ppm) corresponding to the serine anion found in the spectrum of the purple broccoli extract confirm the presence of the IAA conjugate with serine. The ion at *m*/*z* 263.1025 corresponding to the protonated molecular ion of IAA conjugate with serine (exact mass: 263.1026, Δ 0.4 ppm) and the fragment ions at *m*/*z* 130.0650 (exact mass: 130.0651, Δ 0.8 ppm, **2**) diagnostic for the presence of indole ring and 106.0500 (exact mass: 106.0499, Δ 0.9 ppm) corresponding to the serine protonated cation found in the spectrum of the purple broccoli extract in the positive ion mode confirm the presence of the IAA conjugate with serine.

**Figure 7:**
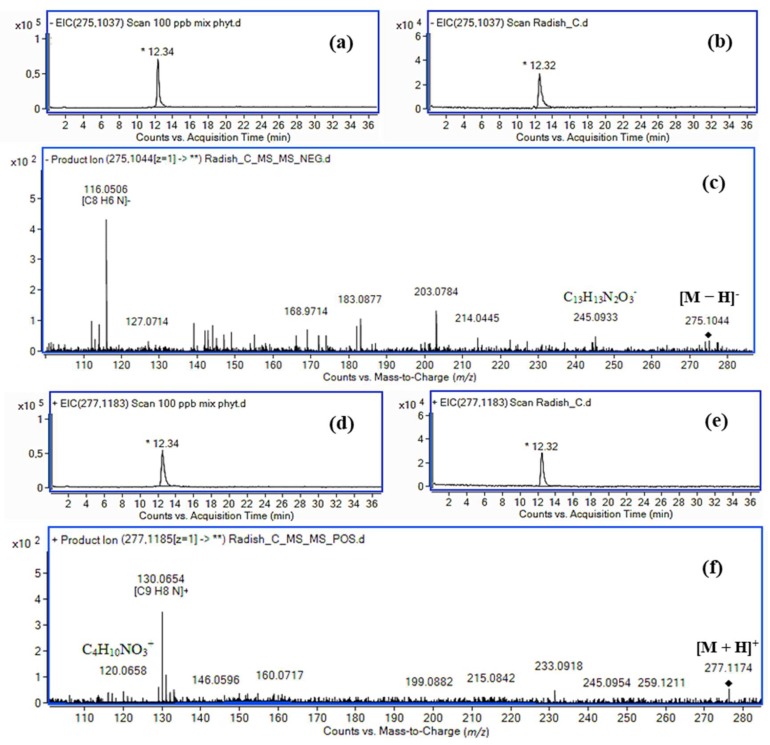
Extracted ion chromatograms for (**a**) a standard solution of IASer-Me, (**b**) a radish extract, and (**c**) an MS/MS spectrum of the extract in negative ionization mode. Extracted ion chromatograms for (**d**) a standard solution of IASer-Me, (**e**) a radish extract, and (**f**) an MS/MS spectrum of the extract in positive ionization mode.

Extracted ion chromatograms for a standard solution of IAA conjugate with serine methyl ester and for the radish extract are presented in Figure 7, along with the MS/MS spectra of the radish extract in negative and positive ionization modes. The molecular ion at *m*/*z* 275.1044 corresponding to the deprotonated molecular ion of IASer-Me (exact mass: 275.1037, Δ 2.5 ppm) and the fragment ions at *m*/*z* 116.0506 (exact mass: 116.0506, Δ 0 ppm, **5**) and 245.0933 (exact mass: 245.0932, Δ 0.4 ppm, **7**) in the negative ion mode suggest the presence of the IAA conjugate with serine methyl ester. The ion at *m*/*z* 277.1174 corresponding to the deprotonated molecular ion of IASer-Me (exact mass: 277.1183, Δ 3.2 ppm) and the fragment ions at *m*/*z* 130.0654 (exact mass: 130.0651, Δ 2.3 ppm) diagnostic for the indole ring and 120.0658 (exact mass: 120.0655, Δ 2.5 ppm) diagnostic for the serine methyl ester protonated cation in the positive ion mode suggest the presence of the IAA conjugate with serine methyl ester in the radish extract.

The compounds identified in the examined cruciferous vegetable extracts applying our methodology are summarized in Table 2. IAA, IAld, and IAAla were detected in all cruciferous vegetables. 4-Cl-IAA was found only in *B. oleracea* var. *rubra* and *R. raphanistrum* subsp. *sativus*. The intermediate in IAA biosynthesis, IAN, was absent only in *E. sativa* and IAM was present in *B. oleracea* var. *italica* cv. Viοlleto and *R. raphanistrum* subsp. *sativus*. IATrp, IAVal, IAPhe, IAVal-Me, and IASer-Me were found in almost all *Brassicaceae* species studied, whereas IATrp-Me and IAMet-Me were absent in all cases. IAMet was detected in *B. oleracea* var. *rubra* and *B. oleracea* var. *capitata*. In addition, conjugates with amino acids tyrosine (*R. raphanistrum* subsp. *sativus*), glycine (in *B. oleracea* var. *capitata*, *R. raphanistrum* subsp. *Sativus*, and *E. sativa*), aspartic acid (*B. rapa* subsp. *rapifera*), and glutamic acid (in *B. rapa* subsp. *rapifera*, *B. oleracea* var. *italica* cv. Calabrese, and *R. raphanistrum* subsp. *sativus*), as well as their corresponding methyl esters, were detected in some cases. It must be noted that indole-3-acetyl-aspartic acid and indole-3-acetyl-glutamic acid dimethyl esters were detected only in *B. oleracea* var. *capitata*. IASer was detected in purple broccoli and radish, whereas IAAla-Me was detected in *B. rapa* subsp. *rapifera*, *B. oleracea* var. *italica* cv. Calabrese, and *R. raphanistrum* subsp. *sativus*.

Up to now, little has been known regarding the spectrum of IAA conjugates present in a given plant tissue, mainly due to the difficulties of their individual analysis. Most of the knowledge is based on the total IAA obtained after hydrolysis of its conjugates. Studies in the dicot *Arabidopsis* have shown a remarkable difference between the total IAA and free IAA, indicating the presence of a considerable amount of IAA conjugates [20]. Since only IAA conjugates with aspartic acid and glutamic acid were determined in that work by gas chromatography–mass spectrometry (GC/MS) at low concentrations, it was concluded that the major part (98%) of amide conjugates remain to be identified [20]. Kowalczyk and Sandberg, using also an indirect GC/MS method, found that the content of IAA conjugates with Leu, Asp, Glu, and Ala did not significantly contribute to the total IAA in *Arabidopsis* [14]. Pěnčík et al. quantified IAA amino acid conjugates with Ala, Asp, Glu, Val, Leu, Phe, and Gly from a higher plant, *Helleborus niger*, using HPLC-MS/MS in positive ion mode and they clearly demonstrated the vast difference (of about four orders of magnitude) between the contents of free IAA and the IAA conjugate with Ala, the least abundant of the quantified conjugates [18]. Thus, the development of methodologies employing techniques such as HRMS will help in the exploration of IAA conjugates. Developing such an approach, we can identify a series of IAA conjugates in cruciferous vegetables. The very low concentrations of IAA conjugates in the plant tissues could be a limitation of our methodology, one that can possibly be overcome if a larger quantity of the lyophilized vegetable in the extraction step is used. 

## 3. Materials and Methods 

### 3.1. General Experimental Procedures

The HRMS spectra were recorded on an Agilent 6530 Quadrupole Time of Flight LC-MS system (UPLC-QToF-MS), with an ESI source, coupled with Agilent 1290 Infinity UPLC system and an autosampler (Agilent Technologies, Santa Clara, CA, USA). Nitrogen was used as the collision gas and electrospray ionization (ESI)—negative and positive mode—was used for the MS experiments. The data acquisition was carried out with Agilent MassHunter software (version B.06.00). The following QToF conditions were used: drying gas, 12 L/min; gas temperature, 300 °C; fragmentor, 170V; skimmer, 65 V; capillary voltage, 4000 V; nebulizer gas, 45 psi; acquisition rate, 1 spectra/s (threshold 200 Abs, 0.01% rel.); MS scan range, 50–1500. For the MS/MS experiments, an auto-MS/MS method was developed with the following parameters: MS/MS acquisition rate, 1 spectra/s (threshold 5 Abs, 0.01% rel.); MS/MS scan range, 50–1500; collision energy slope, 5 V; offset, 2.5 V; preferred charge state, 2, 1, unknown. The mass accuracy of the QToF-MS was calibrated before each analysis using a reference solution for scanning up to *m*/*z* 1500. Mass calibration of the QToF-MS was controlled by constant infusion of a reference mass solution (obtained from Agilent Technologies) into the source of the QToF-MS during the analysis. The ions selected were the reference ions 121.0509 and 922.0098 for positive ESI mode, whereas in negative ESI mode, the selected reference ions were 112.9856 and 1033.9881. The raw data files were processed with Agilent MassHunter Qualitative Analysis software (version B.07.00).

The extraction from cruciferous vegetables was performed using a simple extraction method with 80% MeOH, followed by classical filtration. The simultaneous identification of the studied compounds using UPLC-QToF-MS was conducted in negative and positive ESI mode. Identification was based on retention time relative to that of the standard compound, the accurate mass of ions, and their MS/MS spectra. 

Chromatographic study of the compounds was performed with an Agilent Zorbax C18 (50 × 2.1 mm, 1.8 μm) column (Agilent Technologies, Santa Clara, CA, USA). The mobile phase was ultrapure water–formic acid 0.1% (A) and MeOH–formic acid 0.1% (B) with the following gradient: 0 min: 5% B; 1 min: 5% B; 8.5 min: 95% B; 9.5 min: 95% B; 11.5 min: 5% B; 36.5 min: 5% B. The total run time including column equilibration was 36.5 min. The injection volume was 2 μL and the flow rate was 0.4 mL min^-1^. The column oven temperature was set at 27 °C.

### 3.2. Reagents and Materials

Indole-3-acetic acid was purchased from Sigma-Aldrich Chemical Co. (St. Louis, MO, USA). 4-Chloroindole-3-acetic acid was purchased from Fluorochem Ltd. (Derbyshire, UK). MeOH and formic acid (LC-MS grade) were obtained from Sigma-Aldrich Chemical Co. (St. Louis, MO, USA). All indole amide conjugates were synthesized by a coupling reaction between IAA and the corresponding amino acid methyl esters [36]. IAld was synthesized by Vilsmeier–Haack reaction, from indole and oxalyl chloride in dimethylformamide [39]. IAN was synthesized from IAld by treatment with sodium borohydride and potassium cyanide [40]. IAM was synthesized from hydrolysis of IAN with sodium hydroxide [41]. Ultra-pure water was provided by MilliQ purification system (Millipore Direct-Q, Bedford, MA, USA). A Heidolph 2 rotary evaporator (Heidolph Instruments GmbH & Co. KG, Schwabach, Germany) and Whatman filter paper grade 1 (Whatman Ltd., Maidstone, UK) were used. 

### 3.3. Sampling

Commercial samples of *Brassica oleracea* L. var. *botrytis* L. cv. Zarka, *Brassica oleracea* L. var. *rubra* L., *Brassica oleracea* L. var. *capitata* L., *Brassica oleracea* L. var. *italica* Plenck cv. Calabrese, *Brassica oleracea* L. var. *italica* Plenck cv. Viοlleto, *Raphanus raphanistrum* L. subsp. *sativus* (L.) Domin, *Eruca sativa* (L.) Mill., and *Brassica rapa* L. subsp. *rapifera* Metzg. originated from Chalkida (38°28′02.5″ N 23°38′13.4″ E), Evia County, Greece. All samples were collected from a local producer in February 2017 and they were at the developmental stage of consumption. Leaves from *Brassica oleracea* L. var. *rubra* L., *Brassica oleracea* L. var. *capitata* L., and *Eruca sativa* (L.) Mill., florets of *Brassica oleracea* L. var. *italica* Plenck cv. Calabrese, *Brassica oleracea* L. var. *italica* Plenck cv. Viοlleto, and *Brassica oleracea* L. var. *botrytis* L. cv. Zarka, and roots of *Raphanus raphanistrum* L. subsp. *sativus* (L.) Domin and *Brassica rapa* L. subsp. *rapifera* Metzg. were used for the preparation of extracts. Samples were lyophilized and ground to a fine homogenous powder using mortar and pestle.

### 3.4. Preparation of Extracts 

The preparation of extracts was performed according to the literature [16] with the following modifications: 1 g of dry tissue was extracted at 4 °C for 24 h with 20 mL of 80% MeOH. The solvent was collected, and the residue re-extracted with 20 mL of 80% MeOH for 30 min at 4 °C. The organic layers were combined, dried with 1 g of anhydrous sodium sulfate, and filtered using Whatman filter paper grade 1. After filtration, the extract was evaporated to dryness at 35 °C under reduced vacuum using a rotary evaporator. The residue was dissolved in 1 mL MeOH. A 50 μL amount of the extract was diluted with 50 μL of MeOH and then injected into the LC-MS system.

### 3.5. Standard Solutions

Stock solutions (1000 μg mL^−1^) of IAA, 4-Cl-IAA, IAld, IAN, IAM, indole-3-acetyl-l-alanine methyl ester, indole-3-acetyl-glycine methyl ester, indole-3-acetyl-l-valine methyl ester, indole-3-acetyl-l-tryptophan methyl ester, indole-3-acetyl-l-tyrosine methyl ester, indole-3-acetyl-l-serine methyl ester, indole-3-acetyl-l-phenylalanine methyl ester, indole-3-acetyl-l-methionine methyl ester, indole-3-acetyl-l-aspartic acid dimethyl ester, indole-3-acetyl-l-glutamic acid dimethyl ester, indole-3-acetyl-l-alanine, indole-3-acetyl-l-valine, indole-3-acetyl-glycine, indole-3-acetyl-l-tryptophan, indole-3-acetyl-l-tyrosine, indole-3-acetyl-l-serine, indole-3-acetyl-l-phenylalanine, indole-3-acetyl-l-methionine, indole-3-acetyl-l-aspartic acid, and indole-3-acetyl-l-glutamic acid were prepared in methanol and stored in dark glass containers at −20 °C. From these solutions, working standard solutions were prepared daily by dilution with MeOH. A solution of 10 μg mL^−1^ in MeOH of each compound was used for the full scan and MS/MS experiments.

## 4. Conclusions

We employed high-resolution mass spectrometry as an instrumental technique for the structural elucidation of indole acetic acid major metabolites found in plants. The molecular and fragment ions of IAA and IAA metabolites, particularly of IAA amide conjugates with amino acids bearing a free carboxylic group or a methyl ester group, were described and 25 compounds were identified in *Brassicaceae* using an easy, rapid, and accurate UPLC-QToF-MS analytical protocol in a single run. To the best of our knowledge, this is the first report employing high-resolution mass spectrometry especially for the study of IAA amide conjugates, in cruciferous vegetables. This work will provide a helpful methodology for further study of previously undescribed plant hormone conjugates in plant tissues and could open new pathways to generate a whole-plant organ distribution map of IAA and IAA metabolites depending on the developmental stage of the plant.

## Figures and Tables

**Table 1:** High-resolution mass data in ESI positive and negative ion mode and elemental compositions of the molecular and characteristic fragment ions, mass error ∆, peak intensity, retention time of compounds, and isotopic abundance distribution match.

Compounds	Rt ^1^ (min)	Positive Ion Mode						Negative Ion Mode					
		Ion Species	Elemental Composition	Theoretical Mass (*m*/*z*)	Observed Mass (*m*/*z*) (Mass Error, ∆ ppm)	Peak Intensity	Score (Iso. abund.) ^2^	Ion Species	Elemental Composition	Theoretical Mass (*m*/*z*)	Observed Mass (*m*/*z*) (Mass Error, ∆ (ppm)	Peak Intensity	Score (Iso. abund.) ^2^
Indole-3-acetic acid (IAA)	13.12	[M + H]^+^	C_10_H_10_NO_2_^+^	176.0706	176.0704 (1.1)	1.8 × 10^4^	94.9	[M − H]^−^	C_10_H_8_NO_2_^−^	174.0561	174.0555 (3.4)	4.0 × 10^3^	90.63
		[Μ + Νa]^+^	C_10_H_9_NNaO_2_^+^	198.0525	198.0528 (1.5)	0.6 × 10^4^	90.79	4	C_9_H_8_N^−^	130.0662	130.0656 (4.6)	2.2 × 10^3^	91.21
		2	C_9_H_8_N^+^	130.0651	130.0651 (0)	6.7 × 10^3^	94.76						
4-Chloroindole-3-acetic acid (4-Cl-IAA)	13.93	[M + H]^+^	C_10_H_9_ClNO_2_^+^	210.0316	210.0317 (0.5)	1.4 × 10^5^	99.71		C_10_H_7_ClNO_2_^−^	208.0171	208.0167 (1.9)	8.2 × 10^5^	99.25
		[M + H]^+^	C_10_H_9_ClNO_2_^+^	212.0287	212.0292 (2.4)	0.5 × 10^5^			C_10_H_7_ClNO_2_^−^	210.0142	210.0136 (2.9)	2.9 × 10^5^	
		[Μ + Νa]^+^	C_10_H_8_ClNNaO_2_^+^	232.0136	232.0137 (0.4)	2.1 × 10^5^	99.51		C_9_H_7_ClN^−^	164.0273	164.0271 (1.2)	4.9 × 10^3^	90.86
		[Μ + Νa]^+^	C_10_H_8_ClNNaO_2_^+^	234.0106	234.0110 (1.7)	7.1 × 10^4^			C_9_H_7_ClN^−^	166.0244	166.0246 (1.2)	2.3 × 10^3^	
			C_9_H_7_ClN^+^	164.0252	164.0252 (0)	0.9 × 10^4^	90.06						
			C_9_H_7_ClN^+^	166.0223	166.0231 (4.8)	0.3 × 10^4^							
Indole-3-aldehyde (IAld)	12.82	[M + H]^+^	C_9_H_8_NO^+^	146.06	146.0601 (0.7)	8.3 × 10^5^	99.2	[M − H]^−^	C_9_H_6_NO^−^	144.0455	146.0451 (0.7)	3.1 × 10^6^	98.68
		[Μ + Νa]^+^	C_9_H_7_NNaO^+^	168.042	168.0420 (0)	1.5 × 10^5^	99.88	5	C_8_H_6_N^−^	116.0506	116.0503 (2.6)	7.2 × 10^2^	90.67
		[M + H − CO]^+^	C_8_H_8_N^+^	118.0651	118.0653 (1.7)	8.1 × 10^3^	99.92						
Indole-3-acetonitrile (IAN)	13.38	[M + H]^+^	C_10_H_9_N_2_^+^	157.076	157.0765 (3.2)	6.0 × 10^4^	98.84		C_10_H_7_N_2_^−^	155.0615	155.0615 (0)	4.0 × 10^5^	99.53
		[Μ + Νa]^+^	C_10_H_8_N_2_Na^+^	179.0579	179.0583 (2.2)	7.8 × 10^4^	99.46		C_9_H_6_N^−^	128.0506	128.0508 (2.3)	4.0 × 10^2^	91.46
		2	C_9_H_8_N^+^	130.0651	130.0655 (3.1)	2.6 × 10^2^	98.01						
Indole-3-acetamide (IAM)	12.81	[M + H]^+^	C_10_H_11_N_2_O^+^	175.0866	175.0866 (0)	1.0 × 10^5^	90.6	[M − H]^−^	C_10_H_9_N_2_O^−^	173.072	173.0716 (2.3)	4.2 × 10^4^	90.2
		[Μ + Νa]^+^	C_10_H_10_N_2_NaO^+^	197.0685	197.0683 (1.0)	1.9 × 10^6^	98.9	4	C_9_H_8_N^−^	130.0662	130.0663 (0.8)	3.5 × 10^3^	99.94
		2	C_9_H_8_N^+^	130.0651	130.0650 (0.8)	3.1 × 10^4^	99.8						
Indole-3-acetyl-l-alanine (IAAla)	12.64	[M + H]^+^	C_13_H_15_N_2_O_3_^+^	247.1077	247.1077 (0)	7.9 × 10^5^	99.91	[M − H]^−^	C_13_H_13_N_2_O_3_^−^	245.0932	245.0928 (1.6)	2.3 × 10^5^	99.95
		[Μ + Νa]^+^	C_13_H_14_N_2_NaO_3_^+^	269.0896	269.0896 (0)	8.5 × 10^5^	99.65	[H–Ala–OH − H]^−^	C_3_H_6_NO_2_^−^	88.0404	88.0405 (1.1)	6.4 × 10^3^	98.32
		2	C_9_H_8_N^+^	130.0651	130.0649 (1.5)	6.9 × 10^3^	98.91						
		[H–Ala–OH + H]^+^	C_3_H_8_NO_2_^+^	90.055	90.0550 (0)	1.8 × 10^3^	99.36						
Indole-3-acetyl-l-valine (IAVal)	13.86	[M + H]^+^	C_15_H_19_N_2_O_3_^+^	275.139	275.1391 (0.4)	1.3 × 10^6^	99.75	[M − H]^−^	C_15_H_17_N_2_O_3_^−^	273.1245	273.1240 (1.8)	2.2 × 10^6^	99.6
		[Μ + Νa]^+^	C_15_H_18_N_2_NaO_3_^+^	297.1209	297.1208 (0.3)	2.0 × 10^6^	99.47	[H–Val–OH − H]^−^	C_5_H_10_NO_2_^−^	116.0717	116.0717 (0)	6.0 × 10^3^	90.33
		2	C_9_H_8_N^+^	130.0651	130.0651 (0)	1.2 × 10^4^	98.71						
		[H–Val–OH + H]^+^	C_5_H_12_NO_2_^+^	118.0863	118.0861 (1.7)	3.5 × 10^3^	90.83						
Indole-3-acetyl-l-glycine (IAGly)	11.76	[M + H]^+^	C_12_H_13_N_2_O_3_^+^	233.0921	233.0919 (0.9)	2.5 × 10^5^	99.83	[M − H]^−^	C_12_H_11_N_2_O_3_^−^	231.0775	231.0770 (2.2)	1.3 × 10^6^	99.75
		[Μ + Νa]^+^	C_12_H_12_N_2_NaO_3_^+^	255.074	255.0742 (0.4)	5.3 × 10^5^	99.64	[H–Gly–OH − H]^−^	C_2_H_4_NO_2_^−^	74.0248	74.0248 (0)	5.0 × 10^3^	90.54
		2	C_9_H_8_N^+^	130.0651	130.0649 (1.5)	5.9 × 10^3^	93.33						
		[H–Gly–OH + H]^+^	C_2_H_6_NO_2_^+^	76.0393	76.0393 (0)	4.1 × 10^2^	90.49						
Indole-3-acetyl-l-methionine (IAMet)	13.72	[M + H]^+^	C_15_H_19_N_2_O_3_S^+^	307.1111	307.1113 (0.7)	8.0 × 10^5^	98.87	[M − H]^−^	C_15_H_17_N_2_O_3_S^−^	305.0965	305.0960 (1.6)	2.0 × 10^6^	98.73
		[Μ + Νa]^+^	C_15_H_18_N_2_NaO_3_S^+^	329.093	329.0926 (1.2)	2.0 × 10^6^	98.55	[H–Met–OH − H]^−^	C_5_H_10_NO_2_S^−^	148.0438	148.0438 (0)	5.2 × 10^3^	96.69
		[H–Met–OH + H]^+^	C_5_H_12_NO_2_S^+^	150.0583	150.0583 (0)	9.0 × 10^2^	89.51						
		2	C_9_H_8_N^+^	130.0651	130.0650 (0.8)	3.8 × 10^5^	88.7						
Indole-3-acetyl-l-tryptophan (IATrp)	14.15	[M + H]^+^	C_21_H_20_N_3_O_3_^+^	362.1499	362.1499 (0)	8.2 × 10^5^	99.45	[M − H]^−^	C_21_H_18_N_3_O_3_^−^	360.1354	360.1351 (0.8)	8.0 × 10^5^	98.46
		[Μ + Νa]^+^	C_21_H_19_N_3_NaO_3_^+^	384.1318	384.1319 (0.3)	8.2 × 10^5^	99.8	[H–Trp–OH − H]^−^	C_11_H_11_N_2_O_2_^−^	203.0826	203.0825 (0.5)	5.8 × 10^3^	90.69
		[H–Trp–OH + H]^+^	C_11_H_13_N_2_O_2_^+^	205.0972	205.0972 (0)	0.9 × 10^3^	94.82	5	C_8_H_6_N^−^	116.0506	116.0502 (3.4)	8.1 × 10^2^	89.74
		2	C_9_H_8_N^+^	130.0651	130.0649 (1.5)	4.1 × 10^3^	95.63						
Indole-3-acetyl-l-tyrosine (IATyr)	13.53	[M + H]^+^	C_19_H_19_N_2_O_4_^+^	339.1339	339.1341 (0.6)	2.8 × 10^5^	99.61	[M − H]^−^	C_19_H_17_N_2_O_4_^−^	337.1194	337.1191 (0.9)	1.1 × 10^6^	99.73
		[Μ + Νa]^+^	C_19_H_18_N_2_NaO_4_^+^	361.1159	361.1156 (0.8)	8.5 × 10^5^	99.49	[H–Tyr–OH − H]^−^	C_9_H_10_NO_3_^+^	180.0666	180.0663 (1.7)	4.9 × 10^3^	90.58
		[H–Tyr–OH + H]^+^	C_9_H_12_NO_3_^+^	182.0812	182.0816 (2.2)	2.5 × 10^2^	90.55						
		2	C_9_H_8_N^+^	130.0651	130.0649 (1.5)	1.1 × 10^3^	97.7						
Indole-3-acetyl-l-serine (IASer)	11.29	[M + H]^+^	C_13_H_15_N_2_O_4_^+^	263.1026	263.1028 (0.8)	7.0 × 10^5^	99.85	[M − H]^−^	C_13_H_13_N_2_O_4_^−^	261.0881	261.0871 (3.8)	2.1 × 10^6^	99.69
		[Μ + Νa]^+^	C_13_H_14_N_2_NaO_4_^+^	285.0846	285.0848 (0.7)	1.5 × 10^6^	99.64	[H–Ser–OH − H]^−^	C_3_H_6_NO_3_^+^	104.0353	104.0350 (2.9)	2.2 × 10^4^	99.26
		2	C_9_H_8_N^+^	130.0651	130.0650 (0.8)	4.1 × 10^3^	98.81						
		[H–Ser–OH + H]^+^	C_3_H_8_NO_3_^+^	106.0499	106.0494 (4.7)	9.5 × 10^2^	90.41						
Indole-3-acetyl-l-phenylalanine (IAPhe)	14.36	[M + H]^+^	C_19_H_19_N_2_O_3_^+^	323.139	323.1392 (0.6)	6.1 × 10^5^	98.84	[M − H]^−^	C_19_H_17_N_2_O_3_^−^	321.1245	321.1241 (1.2)	1.7 × 10^6^	99.46
		[Μ + Νa]^+^	C_19_H_18_N_2_NaO_3_^+^	345.1209	345.1210 (0.3)	7.0 × 10^5^	99.42	[H–Phe–OH − H]^−^	C_9_H_10_NO_2_^−^	164.0717	164.0715 (1.2)	7.0 × 10^3^	96.73
		[H–Phe–OH + H]^+^	C_9_H_12_NO_2_^+^	166.0863	166.0863 (0)	1.5 × 10^3^	95.1						
		2	C_9_H_8_N^+^	130.0651	130.0649 (1.5)	5.5 × 10^3^	97.64						
Indole-3-acetyl-l-aspartic acid (IAAsp)	11.73	[M + H]^+^	C_14_H_15_N_2_O_5_^+^	291.0975	291.0979 (1.4)	6.0 × 10^5^	93.49	[M − H]^−^	C_14_H_13_N_2_O_5_^−^	289.083	289.0821 (3.1)	2.3 × 10^6^	99.81
		[Μ + Νa]^+^	C_14_H_14_N_2_NaO_5_^+^	313.0795	313.0795 (0)	1.1 × 10^6^	99.72	[H–Asp–OH − H]^−^	C_4_H_6_NO_4_^−^	132.0302	132.0300 (1.5)	2.2 × 10^3^	90.38
		[H–Asp–OH + H]^+^	C_4_H_8_NO_4_^+^	134.0448	134.0447 (0.7)	1.0 × 10^4^	90.8	[H–Ala–OH − H]^−^	C_3_H_6_NO_2_^−^	88.0404	88.0404 (0)	1.7 × 10^3^	99.04
		2	C_9_H_8_N^+^	130.0651	130.0652 (0.8)	9.3 × 10^4^	91.9						
Indole-3-acetyl-l-glutamic acid (IAGlu)	11.99	[M + H]^+^	C_15_H_17_N_2_O_5_^+^	305.1132	305.1139 (2.3)	5.0 × 10^5^	99.83	[M − H]^−^	C_15_H_15_N_2_O_5_^−^	303.0986	303.0980 (2.0)	2.5 × 10^6^	99.99
		[Μ + Νa]^+^	C_15_H_16_N_2_NaO_5_^+^	327.0951	327.0952 (0.3)	6.9 × 10^5^	99.91	[H–Glu–OH − H]^−^	C_5_H_8_NO_4_^−^	146.0459	146.0459 (0)	1.8 × 10^3^	90.63
		2	C_9_H_8_N^+^	130.0651	130.0651 (0)	9.9 × 10^3^	90.2						
		[H–Clu–OH + H]^+^	C_5_H_10_NO_4_^+^	148.0604	148.0604 (0)	1.2 × 10^3^	90.66						
Indole-3-acetyl-glycine methyl ester (IAGly-Me)	12.64	[M + H]^+^	C_13_H_15_N_2_O_3_^+^	247.1077	247.1084 (2.8)	3.0 × 10^5^	99.92	[M − H]^−^	C_13_H_13_N_2_O_3_^−^	245.0932	245.0923 (3.7)	3.1 × 10^6^	99.62
		[Μ + Νa]^+^	C_13_H_14_N_2_NaO_3_^+^	269.0896	269.0899 (1.1)	7.5 × 10^5^	99.59	5	C_8_H_6_N^−^	116.0506	116.0505 (0.9)	2.7 × 10^3^	99.04
		2	C_9_H_8_N^+^	130.0651	130.0651 (0)	7.8 × 10^3^	99.57						
		[H–Gly–OMe + H]^+^	C_3_H_8_NO_2_^+^	90.055	90.0553 (3.3)	7.0 × 10^3^	99.3						
Indole-3-acetyl-l-alanine methyl ester (IAAla-Me)	13.22	[M + H]^+^	C_14_H_17_N_2_O_3_^+^	261.1234	261.1235 (0.4)	8.2 × 10^5^	98.6	[M − H]^−^	C_14_H_15_N_2_O_3_^−^	259.1088	259.1082 (2.3)	3.2 × 10^6^	99.85
		[Μ + Νa]^+^	C_14_H_16_N_2_NaO_3_^+^	283.1053	283.1053 (0)	1.7 × 10^6^	99.96	5	C_8_H_6_N^−^	116.0506	116.0506 (0)	2.5 × 10^3^	90.82
		2	C_9_H_8_N^+^	130.0651	130.0648 (2.3)	7.2 × 10^3^	99.21						
		[H–Ala–OMe + H]^+^	C_4_H_10_NO_2_^+^	104.0706	104.0703 (2.9)	1.8 × 10^3^	99.34						
Indole-3-acetyl-l-valine methyl ester (IAVal-Me)	14.24	[M + H]^+^	C_16_H_21_N_2_O_3_^+^	289.1547	289.1550 (1.0)	1.5 × 10^6^	99.85	[M − H]^−^	C_16_H_19_N_2_O_3_^−^	287.1401	287.1396 (1.7)	4.82106	99.97
		[Μ + Νa]^+^	C_16_H_20_N_2_NaO_3_^+^	311.1366	311.1368 (0.6)	1.6 × 10^6^	99.95	5	C_8_H_6_N^−^	116.0506	116.0504 (1.7)	3.1 × 10^3^	99.88
		[H–Val–OMe + H]^+^	C_6_H_14_NO_2_^+^	132.1019	132.1017 (1.5)	3.3 × 10^3^	94.29						
		2	C_9_H_8_N^+^	130.0651	130.0651 (0)	9.2 × 10^3^	98.28						
Indole-3-acetyl-l-tryptophan methyl ester (IATrp-Me)	14.43	[M + H]^+^	C_22_H_22_N_3_O_3_^+^	376.1656	376.1660 (1.1)	4.4 × 10^5^	99.67	[M − H]^−^	C_22_H_20_N_3_O_3_^−^	374.151	374.1502 (2.1)	1.4 × 10^6^	99.95
		[Μ + Νa]^+^	C_22_H_21_N_3_NaO_3_^+^	398.1475	398.1477 (0.5)	6.2 × 10^5^	99.45	7	C_13_H_13_N_2_O_3_^−^	245.0932	245.0934 (0.8)	4.6 × 10^3^	97.87
		[H–Trp–OMe + H]^+^	C_12_H_15_N_2_O_2_^+^	219.1128	219.1129 (0.5)	5.1 × 10^2^	90.51	5	C_8_H_6_N^−^	116.0505	116.0502 (2.6)	9.0 × 10^2^	96.53
		2	C_9_H_8_N^+^	130.0651	130.0651 (0)	5.5 × 10^3^	96.39						
Indole-3-acetyl-l-tyrosine methyl ester (IATyr-Me)	13.86	[M + H]^+^	C_20_H_21_N_2_O_4_^+^	353.1496	353.1497 (0.3)	1.1 × 10^6^	99.93	[M − H]^−^	C_20_H_19_N_2_O_4_^−^	351.135	351.1344 (1.7)	1.3 × 10^6^	99.65
		[Μ + Νa]^+^	C_20_H_20_N_2_NaO_4_^+^	375.1315	375.1321 (1.6)	6.5 × 10^5^	99.48	7	C_13_H_13_N_2_O_3_^−^	245.0932	245.0933 (0.4)	4.9 × 10^3^	93.52
		[H–Tyr–-OMe + H]^+^	C_10_H_14_NO_3_^+^	196.0968	196.0970 (1.0)	1.4 × 10^3^	97.44	5	C_8_H_6_N^−^	116.0505	116.0503 (1.7)	9.8 × 10^2^	95.36
		2	C_9_H_8_N^+^	130.0651	130.0650 (0.8)	4.5 × 10^3^	99.62						
Indole-3-acetyl-l-serine methyl ester (IASer-Me)	12.34	[M + H]^+^	C_14_H_17_N_2_O_4_^+^	277.1183	275.1186 (1.1)	5.6 × 10^5^	99.76	[M − H]^−^	C_14_H_15_N_2_O_4_^−^	275.1037	275.1028 (3.3)	2.0 × 10^6^	99.72
		[Μ + Νa]^+^	C_14_H_16_N_2_NaO_4_^+^	299.1002	299.1001 (0.3)	1.1 × 10^6^	90.66	7	C_13_H_13_N_2_O_3_^−^	245.0932	245.0927 (1.2)	8.0 × 10^5^	90.7
		2	C_9_H_8_N^+^	130.0651	130.0656 (3.8)	3.0 × 10^3^	94.54	5	C_8_H_6_N^−^	116.0506	116.0504 (1.7)	2.0 × 10^3^	94.39
		[H–Ser–OMe + H]^+^	C_4_H_10_NO_3_^+^	120.0655	120.0650 (4.2)	1.0 × 10^3^	99.18						
Indole-3-acetyl-l-phenylalanine methyl ester (IAPhe-Me)	14.56	[M + H]^+^	C_20_H_21_N_2_O_3_^+^	337.1547	337.1547 (0)	7.9 × 10^5^	98.99	[M − H]^−^	C_20_H_19_N_2_O_3_^−^	335.1401	335.1395 (1.8)	2.7 × 10^6^	99.99
		[Μ + Νa]^+^	C_20_H_20_N_2_NaO_3_^+^	359.1366	359.1366 (0)	1.2 × 10^6^	99.08	5	C_8_H_6_N^+^	116.0506	116.0503 (2.6)	1.5 × 10^3^	92.53
		[H–Phe–OMe + H]^+^	C_10_H_14_NO_2_^+^	180.1019	180.1019 (0)	2.4 × 10^3^	90.6						
		2	C_9_H_8_N^+^	130.0651	130.0655 (3.1)	6.1 × 10^3^	97.34						
Indole-3-acetyl-l-methionine methyl ester (IAMet-Me)	14.04	[M + H]^+^	C_16_H_21_N_2_O_3_S^+^	321.1276	321.1273 (1.9)	6.0 × 10^5^	98.68	[M − H]^−^	C_16_H_19_N_2_O_3_S^−^	319.1122	319.1117 (1.6)	3.2 × 10^6^	98.55
		[Μ + Νa]^+^	C_16_H_20_N_2_NaO_3_S^+^	343.1087	343.1088 (0.3)	8.1 × 10^5^	98.42	5	C_8_H_6_N^−^	116.0506	116.0506 (0)	3.3 × 10^3^	99.8
		[H–Met–OMe + H]^+^	C_6_H_14_NO_2_S^+^	164.074	164.0739 (0.6)	1.5 × 10^3^	98.04						
		2	C_9_H_8_N^+^	130.0651	130.0651 (0)	6.0 × 10^3^	99.42						
Indole-3-acetyl-l-aspartic acid dimethyl ester (IAAsp-Me_2_)	13.31	[M + H]^+^	C_16_H_19_N_2_O_5_^+^	319.1288	319.1288 (0)	1.9 × 10^6^	99.81	[M − H]^−^	C_16_H_17_N_2_O_5_^−^	317.1143	317.1140 (0.9)	2.8 × 10^6^	99.81
		[Μ + Νa]^+^	C_16_H_18_N_2_NaO_5_^+^	341.1108	341.1106 (0.6)	4.5 × 10^6^	100	[IAM − H]^−^	C_10_H_9_N_2_O^−^	173.072	173.0723 (1.7)	3.6 × 10^3^	99.09
		[H–Asp-(OMe)_2_ + H]^+^	C_6_H_12_NO_4_^+^	162.0761	162.0766 (3.1)	2.2 × 10^3^	90.56	5	C_8_H_6_N^−^	116.0506	116.0507 (0.9)	5.3 × 10^2^	90.9
		2	C_9_H_8_N^+^	130.0651	130.0652 (0.8)	5.2 × 10^3^	98.94						
Indole-3-acetyl-l-glutamic acid dimethyl ester (IAGlu-Me_2_)	13.5	[M + H]^+^	C_17_H_21_N_2_O_5_^+^	333.1445	333.1441 (1.2)	1.1 × 10^6^	99.8	[M − H]^−^	C_17_H_19_N_2_O_5_^−^	331.1299	331.1290 (2.7)	2.4 × 10^6^	99.89
		[Μ + Νa]^+^	C_17_H_20_N_2_NaO_5_^+^	355.1264	355.1259 (1.4)	1.9 × 10^6^	99.97	7	C_13_H_13_N_2_O_3_^−^	245.0932	245.0932 (0)	1.9 × 10^3^	90.37
		[H–Glu–(OMe)_2_ + H]^+^	C_7_H_14_NO_4_^+^	176.0917	176.0920 (1.7)	1.8 × 10^3^	96.24	4	C_9_H_6_N^−^	130.0661	130.0662 (0.8)	4.7 × 10^2^	90.47
		2	C_9_H_8_N^+^	130.0651	10.0651 (0)	6.9 × 10^3^	98.32	5	C_8_H_6_N^−^	116.0506	116.0503 (2.6)	1.5 × 10^3^	90.35

^1^ Rt, Retention time; ^2^ Score (Iso. abund.), Isotopic abundance distribution match (a measure of the probability that the distribution of isotope abundance ratios calculated for the formula matches the measured data).

**Table 2:** IAA, IAA metabolites, and IAA amide conjugates with acid amino in eight members of the *Brassicaceae* family.

Compounds	*B. oleracea* var. *capitata*	*B. oleracea* var. *rubra*	*B. rapa* subsp. *rapifera*	*B. oleracea* var. *botrytis* cv. Zarka	*B. oleracea* var. *italica* cv. Calabrese	*B. oleracea* var. *italica* cv. Viοlleto	*R. raphanistrum* subsp. *sativus*	*E. sativa*
IAA	+	+	+	+	+	+	+	+
4-Cl-IAA	−	+	−	−	−	−	+	−
IAld	+	+	+	+	+	+	+	+
IAN	+	+	+	+	+	+	+	−
IAM	−	−	−	−	−	+	+	−
IAAla	+	+	+	+	+	+	+	+
IAVal	+	−	+	+	+	+	+	−
IAGly	+	−	−	−	−	−	+	+
IAMet	+	+	−	−	−	−	−	−
IATrp	+	+	+	+	+	+	+	−
IATyr	−	−	−	−	−	−	+	−
IASer	−	−	−	−	−	+	+	−
IAPhe	+	+	+	−	+	+	+	−
IAAsp	−	−	+	−	−	−	−	−
IAGlu	−		+	−	+	−	+	−
IAGly-Me	+	+	+	+	+	−	+	−
IAAla-Me	−	−	+	−	+	−	+	−
IAVal-Me	+	+	+	−	+	+	+	+
IATrp-Me	−	−	−	−	−	−	−	−
IATyr-Me	+	−	+	−	−	+	+	+
IASer-Me	+	+	+	−	+	+	+	+
IAPhe-Me	+	−	−	−	−	−	−	−
IAMet-Me	−	−	−	−	−	−	−	−
IAAsp-(Me)_2_	+	−	−	−	−	−	−	−
IAAGlu-(Me)_2_	+	−	−	−	−	−	−	−

## Supplementary Materials

The following are available online. Mass spectra of studied compounds, extracted ion chromatograms of studied compounds, and MS data of the identification of studied compounds in *Brassicaceae* are given in the Supplemental Materials section available with this paper.
