# Supplementary material for: Identification of Auxin Metabolites in Brassicaceae by Ultra-Performance Liquid Chromatography Coupled with High-Resolution Mass Spectrometry
Source: Molecules. 2019 Jul 18;24(14):2615. doi: 10.3390/molecules24142615 (PMC6680779; doi:10.3390/molecules24142615)
Supplement: Supplementary file 1 [file molecules-24-02615-s001.pdf]

Supplemental Materials

for

Identification of auxin metabolites in Brassicaceae by ultra-pressure liquid chromatography coupled with high resolution mass spectrometry

Panagiota-Kyriaki Revelou, Maroula G. Kokotou, Violetta Constantinou-Kokotou*

Chemical Laboratories, Department of Food Science and Human Nutrition, Agricultural University of Athens, Iera odos 75, Athens 11855, Greece

Contents

Fig. S1 Full scan (a) and MS/MS spectra (b) of indole-3-acetic acid under negative ESI mode. Full scan (c) and MS/MS spectra (d) of indole-3-acetic acid under positive ESI mode

Fig. S2 Full scan (a) and MS/MS spectra (b) of 4-chloroindole-3-acetic acid under negative ESI mode. Full scan (c) and MS/MS spectra (d) of 4-chloroindole-3-acetic acid under positive ESI mode

Fig. S3 Full scan (a) and MS/MS spectra (b) of indole-3-aldehyde under negative ESI mode. Full scan (c) and MS/MS spectra (d) of indole-3-aldehyde under positive ESI mode

Fig. S4 Full scan (a) and MS/MS spectra (b) of indole-3-acetonitrile under negative ESI mode. Full scan (c) and MS/MS spectra (d) of indole-3-acetonitrile under positive ESI mode

Fig. S5 Full scan (a) and MS/MS spectra (b) of indole-3-acetamide under negative ESI mode. Full scan (c) and MS/MS spectra (d) of indole-3-acetamide under positive ESI mode

Fig. S6 Full scan (a) and MS/MS spectra (b) of indole-3-acetyl-L-alanine under negative ESI mode. Full scan (c) and MS/MS spectra (d) of indole-3-acetyl-L-alanine under positive ESI mode

Fig. S7 Full scan (a) and MS/MS spectra (b) of indole-3-acetyl-L-alanine methyl ester under negative ESI mode. Full scan (c) and MS/MS spectra (d) of indole-3-acetyl-L-alanine methyl ester under positive ESI mode

Fig. S8 Full scan (a) and MS/MS spectra (b) of indole-3-acetyl-L-glutamic acid under negative ESI mode. Full scan (c) and MS/MS spectra (d) of indole-3-acetyl-L-glutamic acid under positive ESI mode

Fig. S9 Full scan (a) and MS/MS spectra (b) of indole-3-acetyl-L-glutamic acid dimethyl ester under negative ESI mode. Full scan (c) and MS/MS spectra (d) of indole-3-acetyl-L-glutamic acid dimethyl ester under positive ESI mode

Fig. S10 Full scan (a) and MS/MS spectra (b) of indole-3-acetyl-glycine under negative ESI mode. Full scan (c) and MS/MS spectra (d) of indole-3-acetyl-glycine under positive ESI mode

Fig. S11 Full scan (a) and MS/MS spectra (b) of indole-3-acetyl-glycine methyl ester under negative ESI mode. Full scan (c) and MS/MS spectra (d) of indole-3-acetyl-glycine methyl ester under positive ESI mode

Fig. S12 Full scan (a) and MS/MS spectra (b) of indole-3-acetyl-L-methionine under negative ESI mode. Full scan (c) and MS/MS spectra (d) of indole-3-acetyl-L-methionine under positive ESI mode

Fig. S13 Full scan (a) and MS/MS spectra (b) of indole-3-acetyl-L-methionine methyl ester under negative ESI mode. Full scan (c) and MS/MS spectra (d) of indole-3-acetyl-L-methionine methyl ester under positive ESI mode

Fig. S14 Full scan (a) and MS/MS spectra (b) of indole-3-acetyl-L-phenylalanine under negative ESI mode. Full scan (c) and MS/MS spectra (d) of indole-3-acetyl-L-phenylalanine under positive ESI mode

Fig. S15 Full scan (a) and MS/MS spectra (b) of indole-3-acetyl-L-phenylalanine methyl ester under negative ESI mode. Full scan (c) and MS/MS spectra (d) of indole-3-acetyl-L-phenylalanine methyl ester under positive ESI mode

Fig. S16 Full scan (a) and MS/MS spectra (b) of indole-3-acetyl-L-tryptophan under negative ESI mode. Full scan (c) and MS/MS spectra (d) of indole-3-acetyl-L-tryptophan under positive ESI mode

Fig. S17 Full scan (a) and MS/MS spectra (b) of indole-3-acetyl-L-tryptophan methyl ester under negative ESI mode. Full scan (c) and MS/MS spectra (d) of indole-3-acetyl-L-tryptophan methyl ester under positive ESI mode

Fig. S18 Full scan (a) and MS/MS spectra (b) of indole-3-acetyl-L-tyrosine under negative ESI mode. Full scan (c) and MS/MS spectra (d) of indole-3-acetyl-L-tyrosine under positive ESI mode

Fig. S19 Full scan (a) and MS/MS spectra (b) of indole-3-acetyl-L-tyrosine methyl ester under negative ESI mode. Full scan (c) and MS/MS spectra (d) of indole-3-acetyl-L-tyrosine methyl ester under positive ESI mode

Fig. S20 Full scan (a) and MS/MS spectra (b) of indole-3-acetyl-L-valine under negative ESI mode. Full scan (c) and MS/MS spectra (d) of indole-3-acetyl-L-valine under positive ESI mode

Fig. S21 Full scan (a) and MS/MS spectra (b) of indole-3-acetyl-L-valine methyl ester under negative ESI mode. Full scan (c) and MS/MS spectra (d) of indole-3-acetyl-L-valine methyl ester under positive ESI mode

Fig. S22 Full scan (a) and MS/MS spectra (b) of indole-3-acetyl-L-aspartic acid under negative ESI mode. Full scan (c) and MS/MS spectra (d) of indole-3-acetyl-L-aspartic acid under positive ESI mode

Fig. S23 Full scan (a) and MS/MS spectra (b) of indole-3-acetyl-L-aspartic acid dimethyl ester under negative ESI mode. Full scan (c) and MS/MS spectra (d) of indole-3-acetyl-L-aspartic acid dimethyl ester under positive ESI mode

Fig. S24 Extracted ion chromatograms of studied compounds in negative ESI mode

Table S1 MS data of the identification of studied compounds in *B. oleracea* var. *capitata*

Table S2 MS data of the identification of studied compounds in *B. oleracea* var. *rubra*

Table S3 MS data of the identification of studied compounds in *B. rapa* subsp. *rapifera*

Table S4 MS data of the identification of studied compounds in *B. oleracea* var. *botrytis* cv. zarka

Table S5 MS data of the identification of studied compounds in *B. oleracea* var. *italica* cv. calabrese

Table S6 MS data of the identification of studied compounds in *B. oleracea* var. *italica* cv. violleto

Table S7 MS data of the identification of studied compounds in *R. raphanistrum* subsp. *sativus*

Table S8 MS data of the identification of studied compounds in *E. sativa*

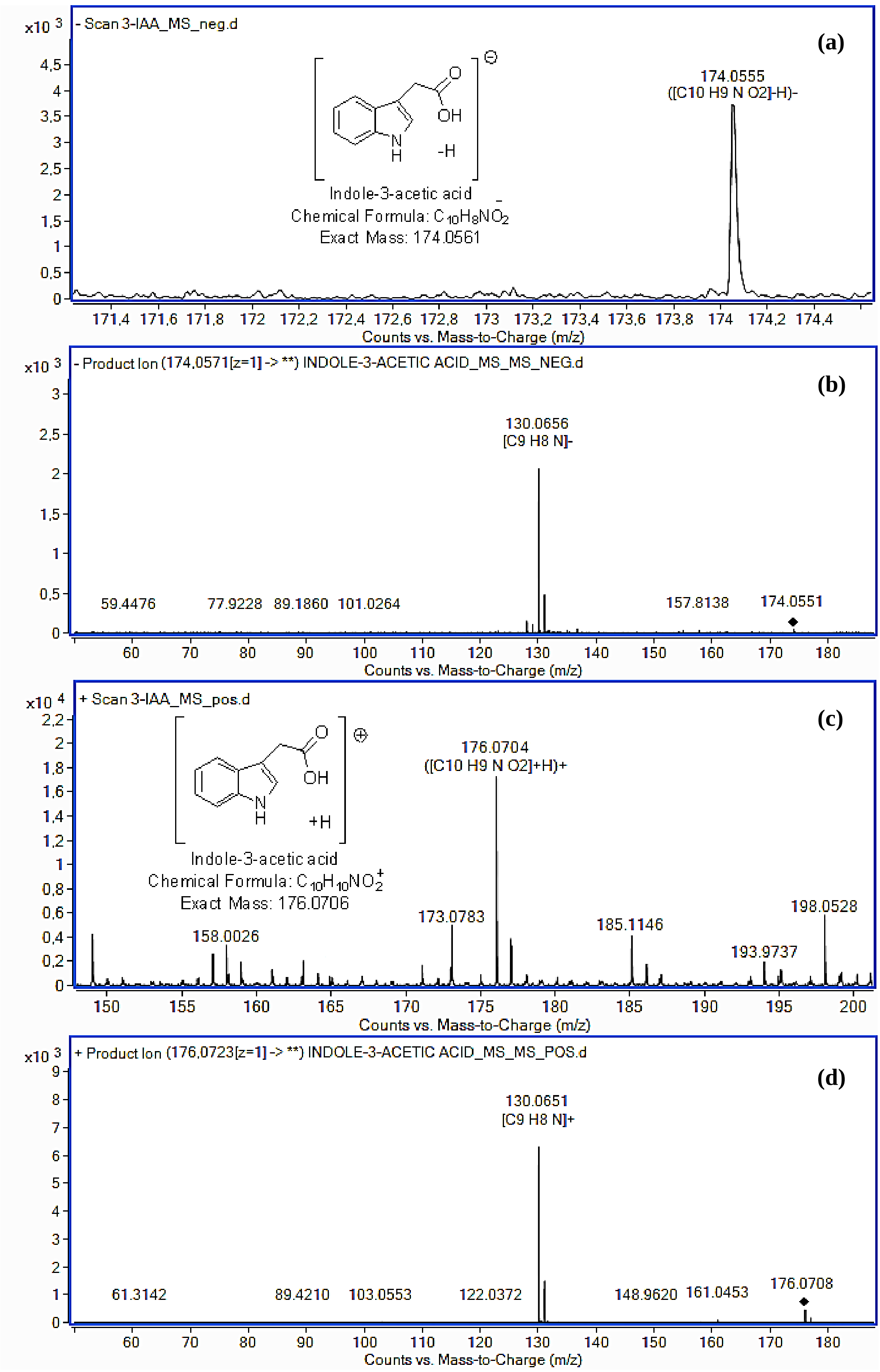

Fig. S1 Full scan (a) and MS/MS spectra (b) of indole-3-acetic acid under negative ESI mode. Full scan (c) and MS/MS spectra (d) of indole-3-acetic acid under positive ESI mode

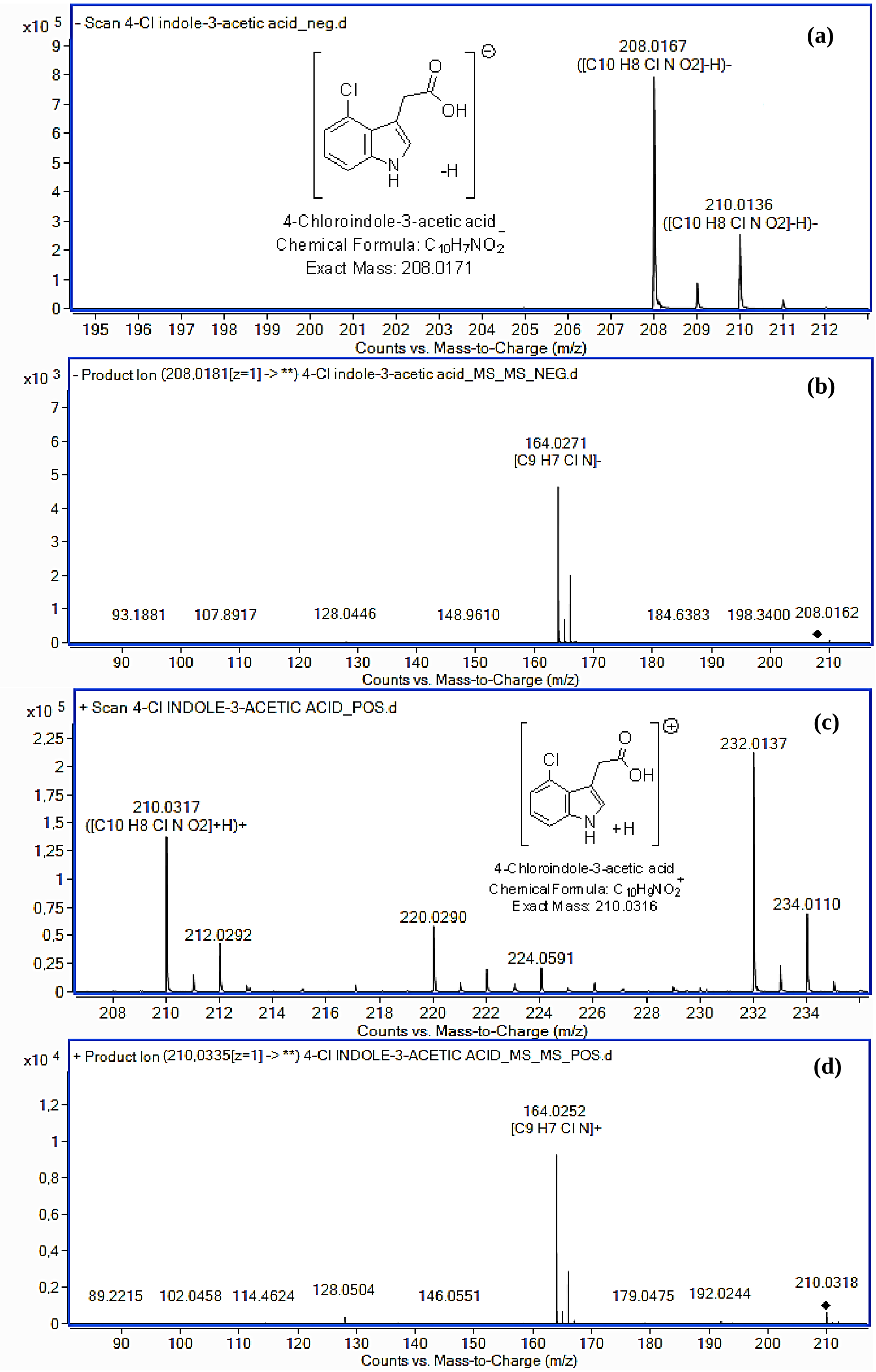

Fig. S2 Full scan (a) and MS/MS spectra (b) of 4-chloroindole-3-acetic acid under negative ESI mode. Full scan (c) and MS/MS spectra (d) of 4-chloroindole-3-acetic acid under positive ESI mode

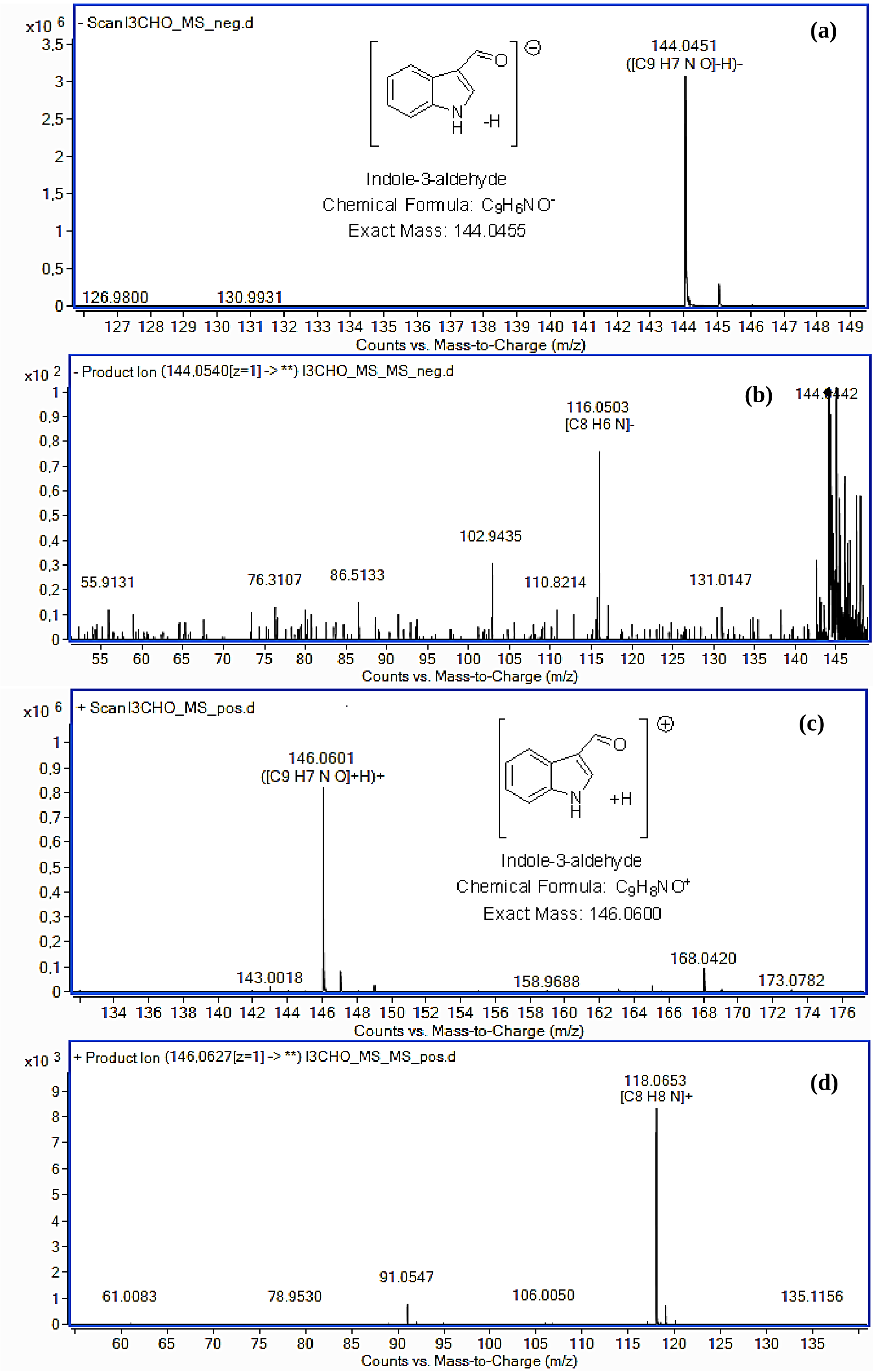

Fig. S3 Full scan (a) and MS/MS spectra (b) of indole-3-aldehyde under negative ESI mode. Full scan (c) and MS/MS spectra (d) of indole-3-aldehyde under positive ESI mode

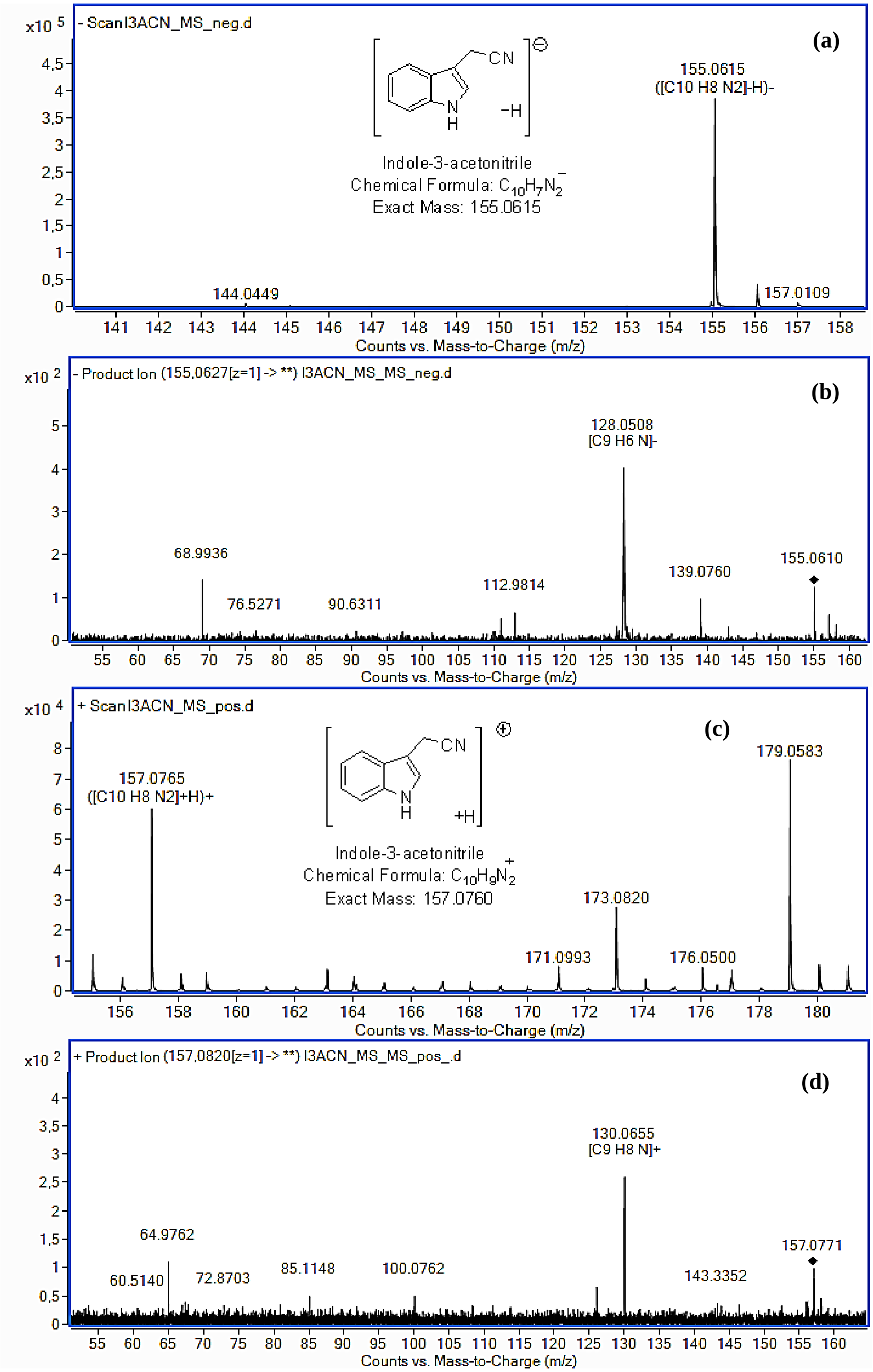

Fig. S4 Full scan (a) and MS/MS spectra (b) of indole-3-acetonitrile under negative ESI mode. Full scan (c) and MS/MS spectra (d) of indole-3-acetonitrile under positive ESI mode

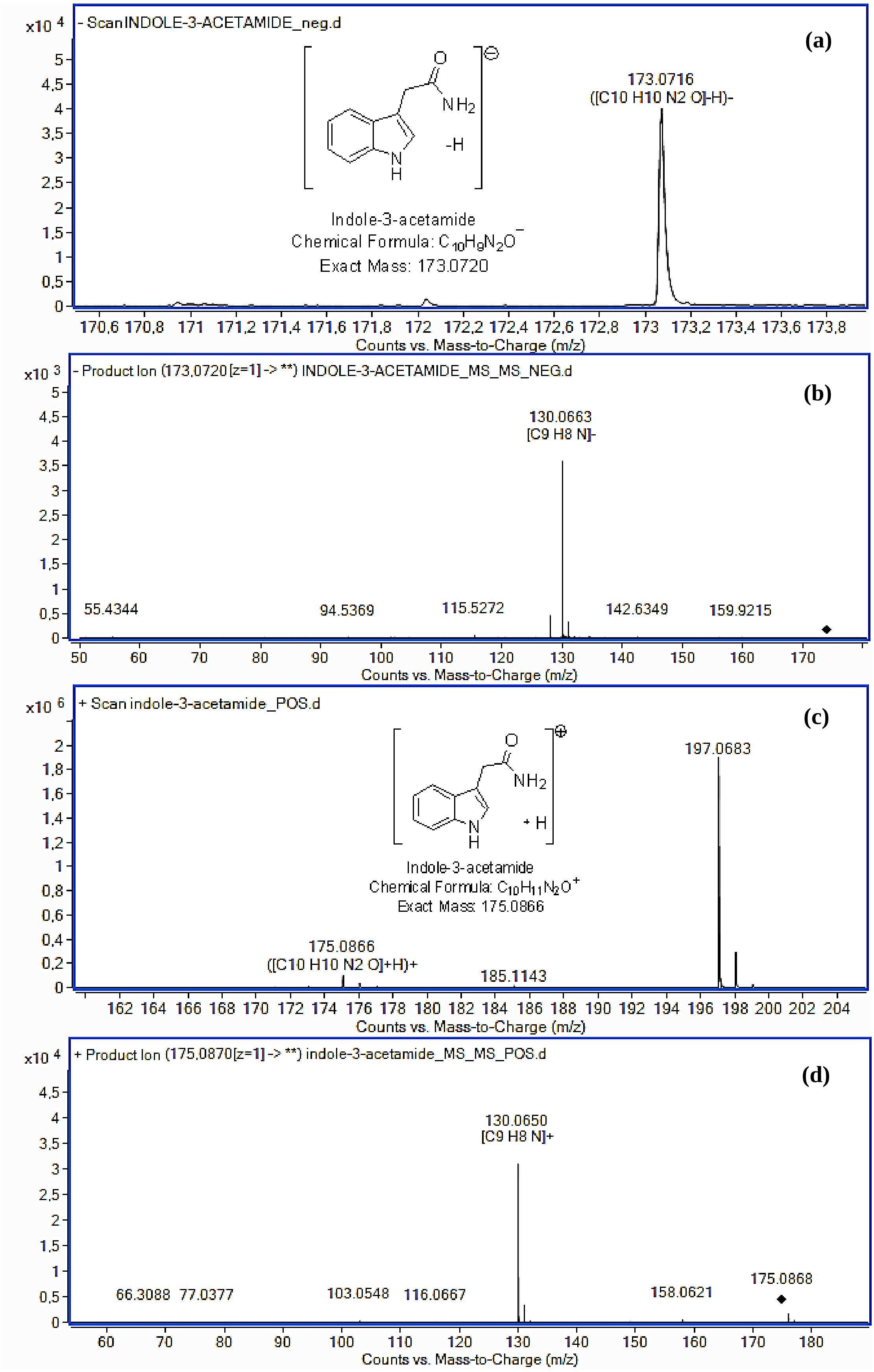

Fig. S5 Full scan (a) and MS/MS spectra (b) of indole-3-acetamide under negative ESI mode. Full scan (c) and MS/MS spectra (d) of indole-3-acetamide under positive ESI mode

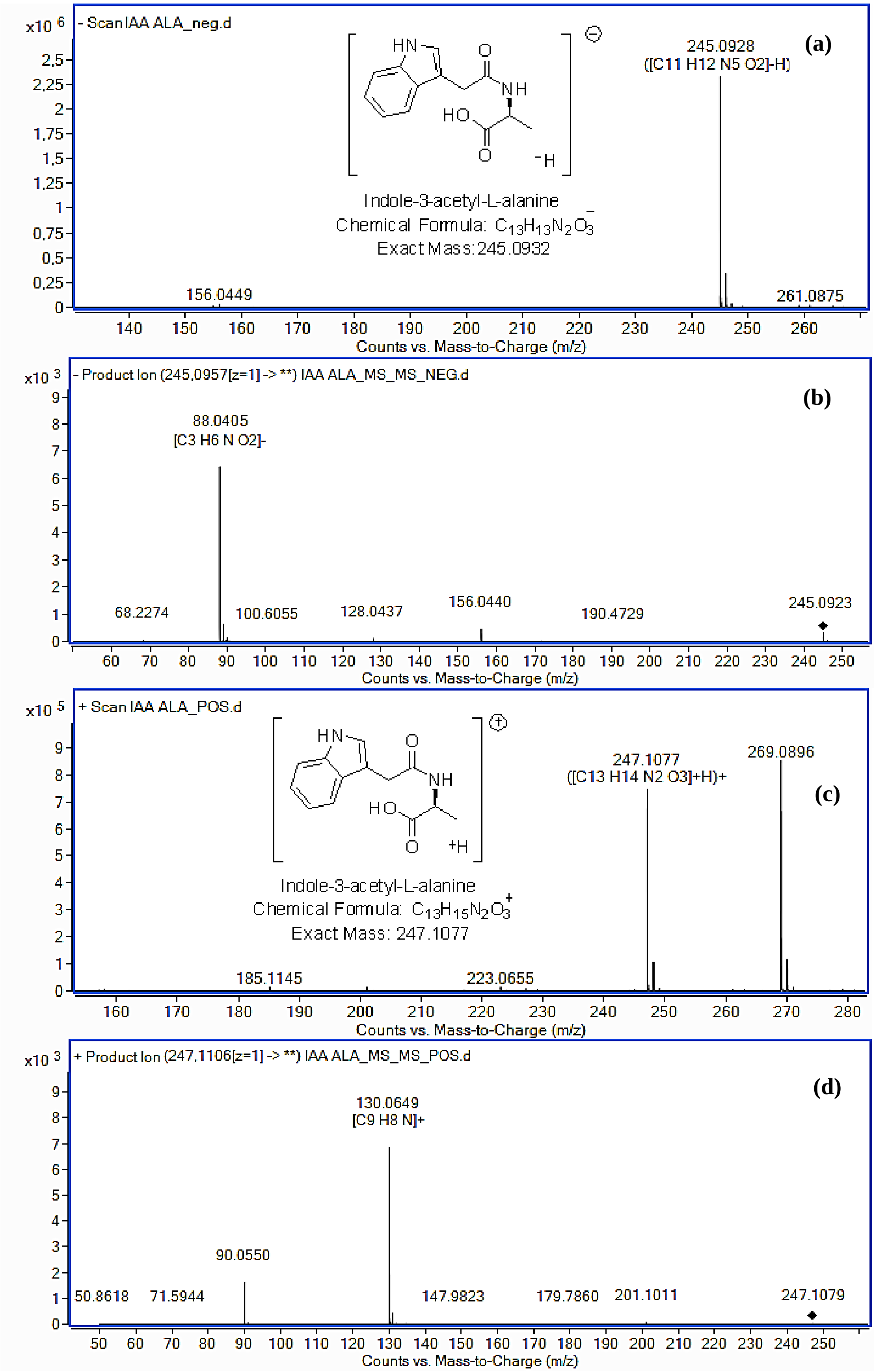

Fig. S6 Full scan (a) and MS/MS spectra (b) of indole-3-acetyl-L-alanine under negative ESI mode. Full scan (c) and MS/MS spectra (d) of indole-3-acetyl-L-alanine under positive ESI mode

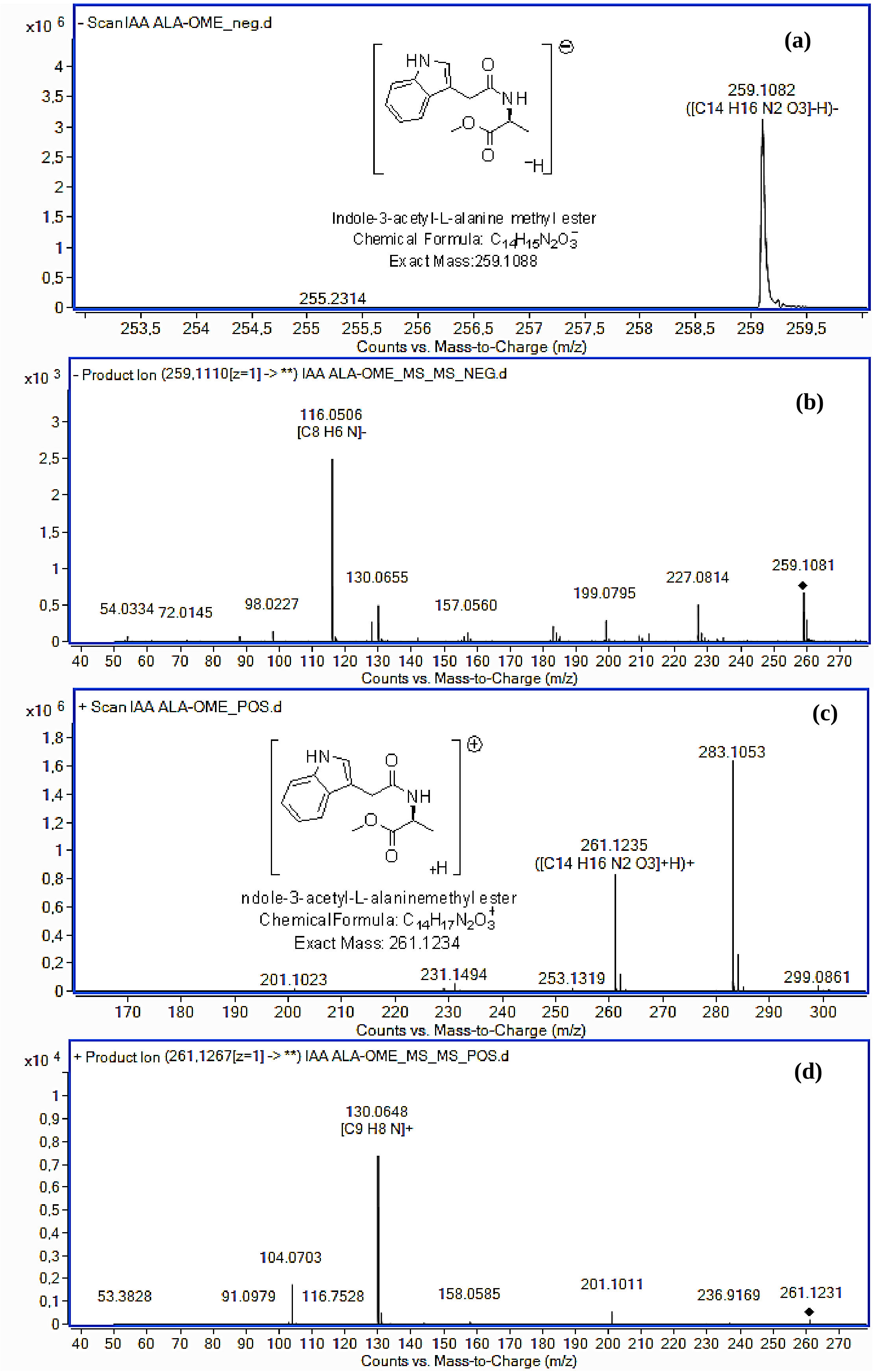

Fig. S7 Full scan (a) and MS/MS spectra (b) of indole-3-acetyl-L-alanine methyl ester under negative ESI mode. Full scan (c) and MS/MS spectra (d) of indole-3-acetyl-L-alanine methyl ester under positive ESI mode

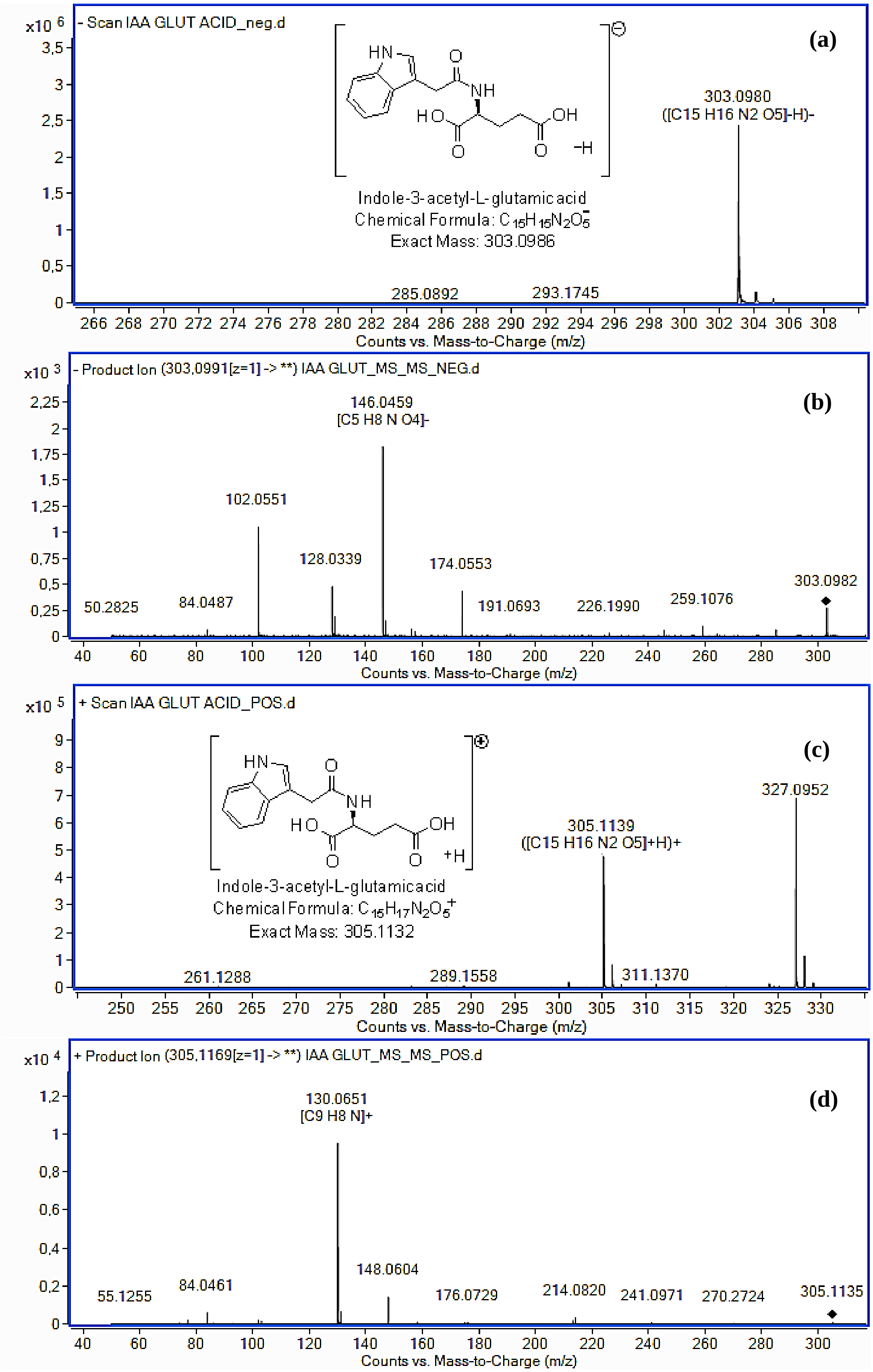

Fig. S8 Full scan (a) and MS/MS spectra (b) of indole-3-acetyl-L-glutamic acid under negative ESI mode. Full scan (c) and MS/MS spectra (d) of indole-3-acetyl-L-glutamic acid under positive ESI mode

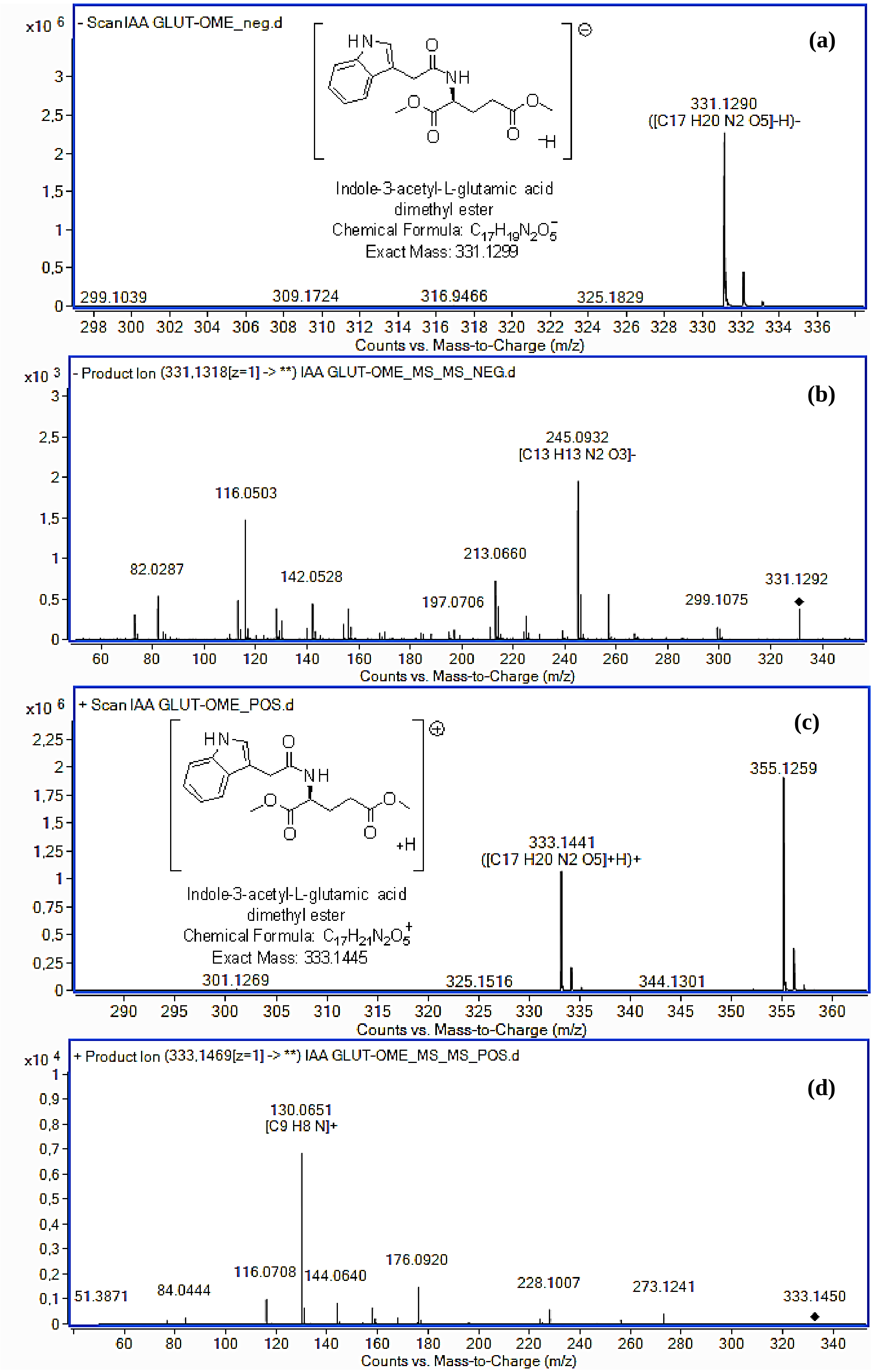

Fig. S9 Full scan (a) and MS/MS spectra (b) of indole-3-acetyl-L-glutamic acid dimethyl ester under negative ESI mode. Full scan (c) and MS/MS spectra (d) of indole-3-acetyl-L-glutamic acid dimethyl ester under positive ESI mode

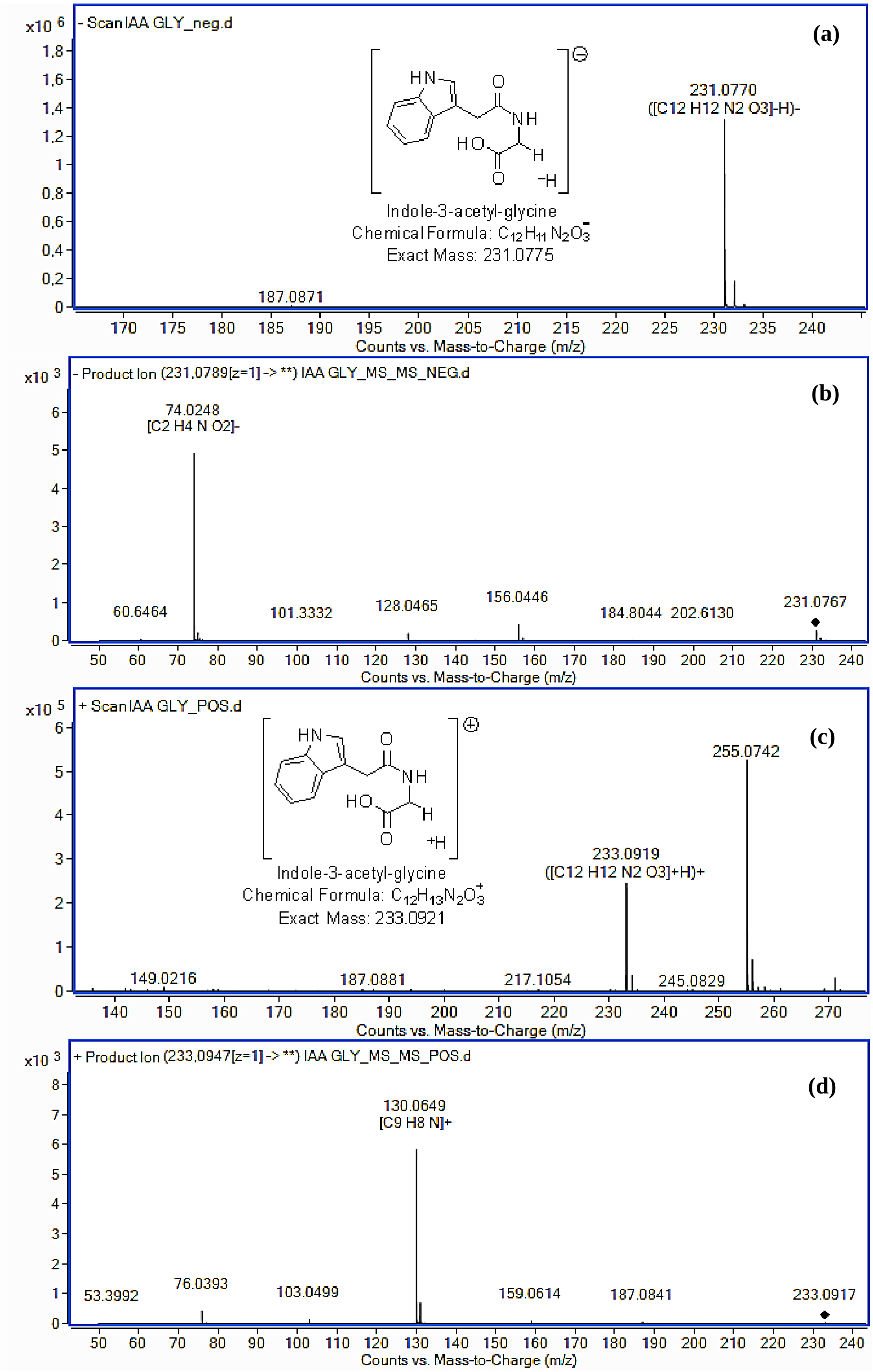

Fig. S10 Full scan (a) and MS/MS spectra (b) of indole-3-acetyl-glycine under negative ESI mode. Full scan (c) and MS/MS spectra (d) of indole-3-acetyl-glycine under positive ESI mode

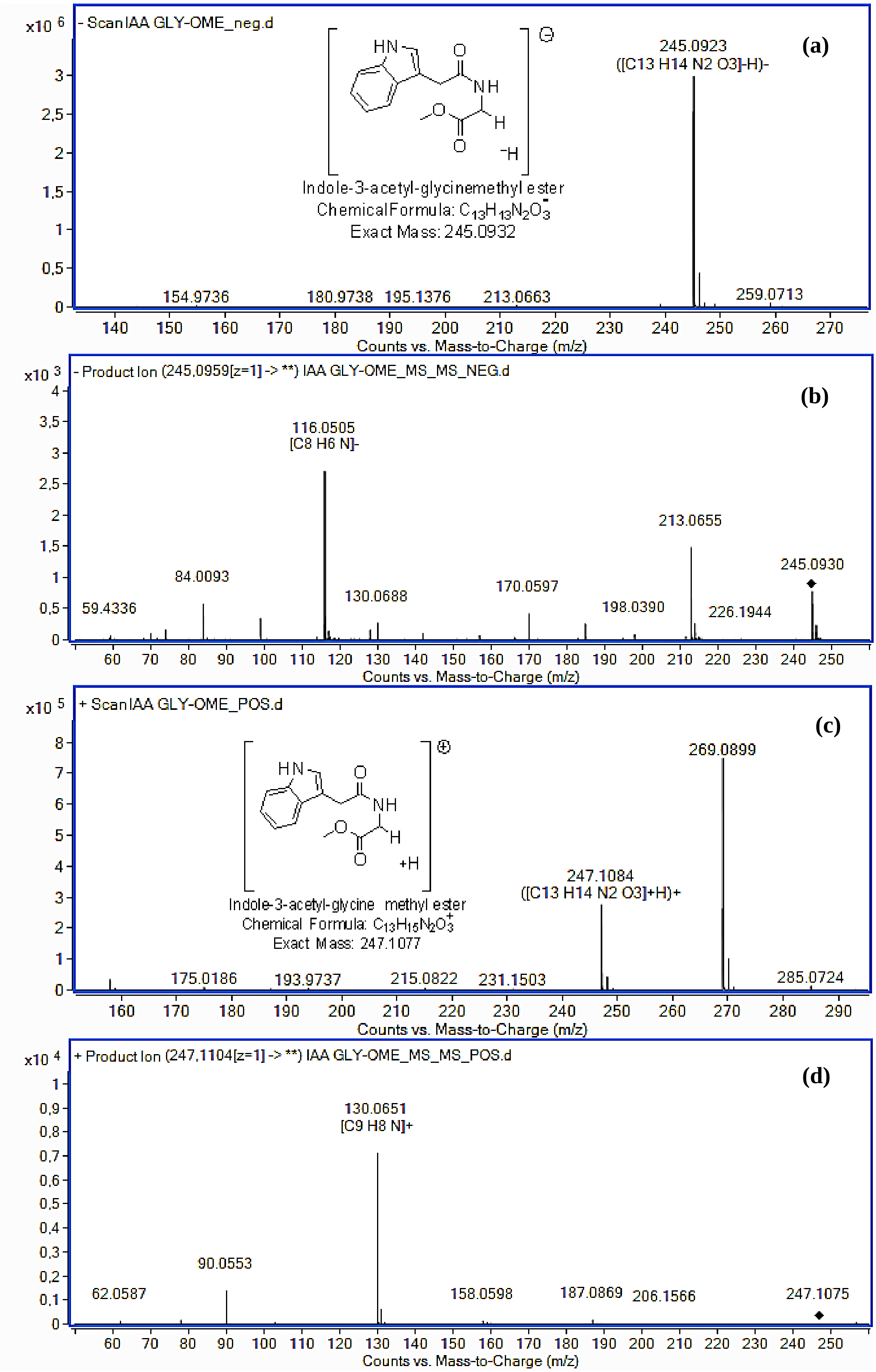

Fig. S11 Full scan (a) and MS/MS spectra (b) of indole-3-acetyl-glycine methyl ester under negative ESI mode. Full scan (c) and MS/MS spectra (d) of indole-3-acetyl-glycine methyl ester under positive ESI mode

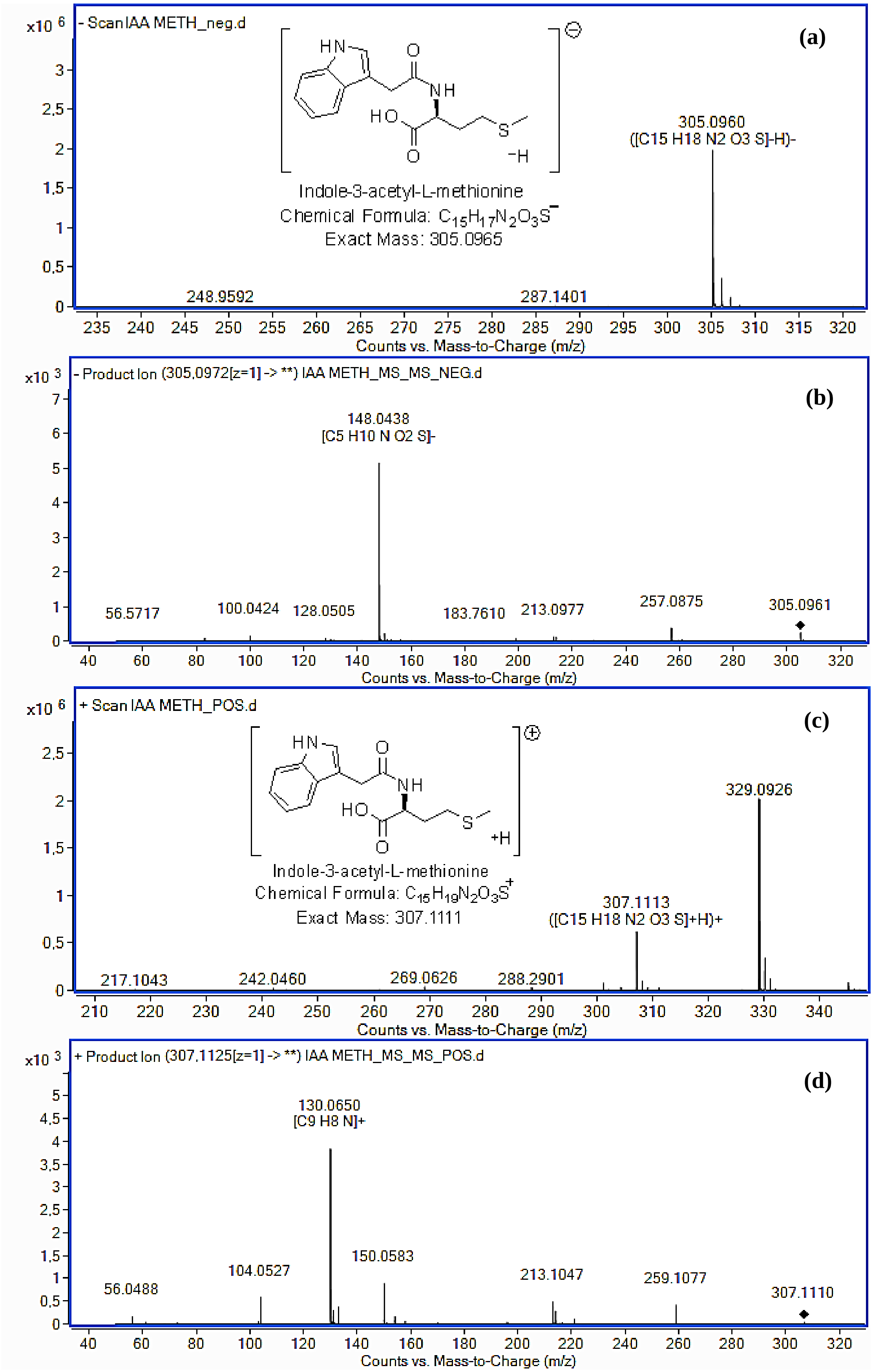

Fig. S12 Full scan (a) and MS/MS spectra (b) of indole-3-acetyl-L-methionine under negative ESI mode. Full scan (c) and MS/MS spectra (d) of indole-3-acetyl-L-methionine under positive ESI mode

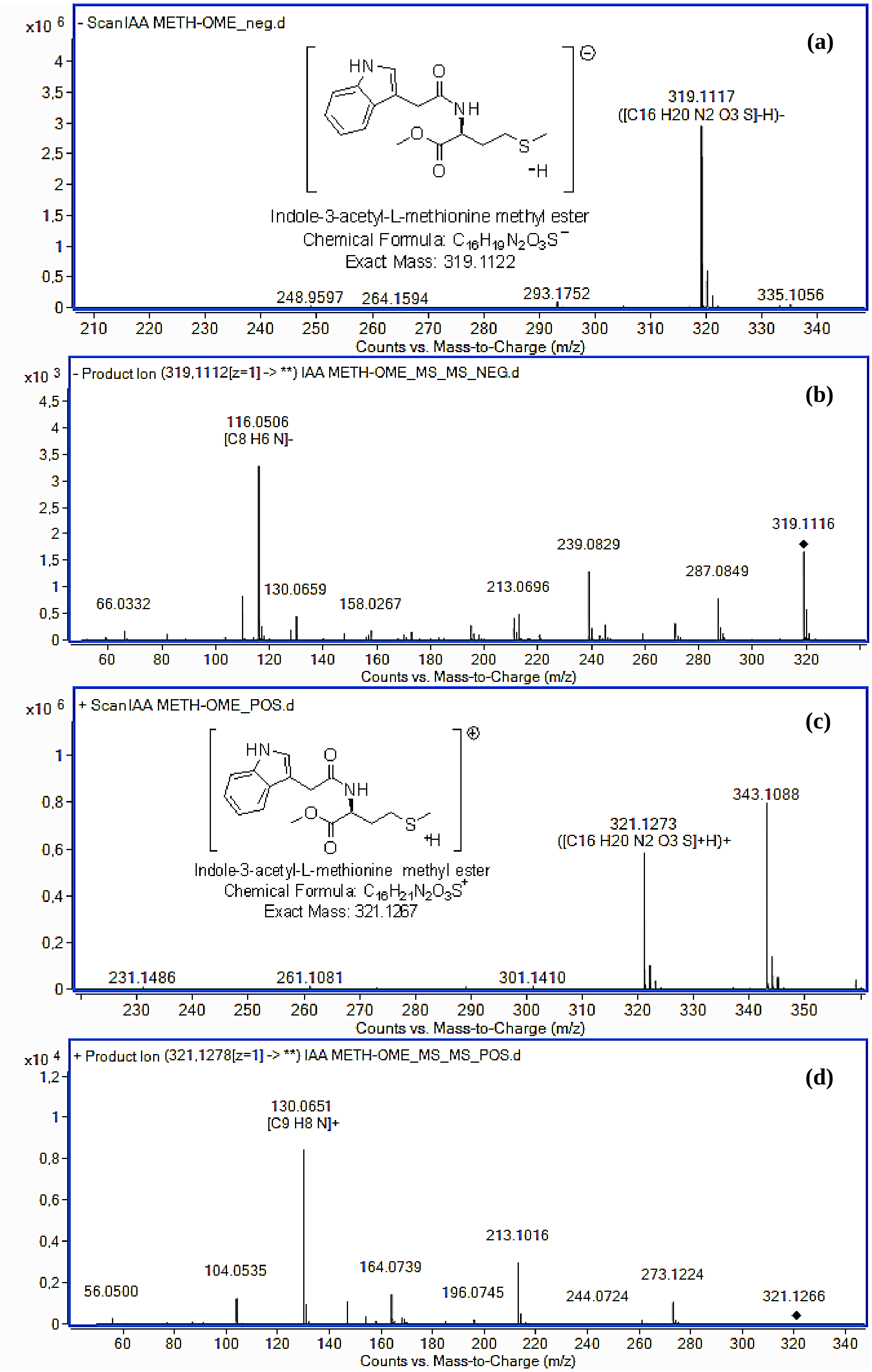

Fig. S13 Full scan (a) and MS/MS spectra (b) of indole-3-acetyl-L-methionine methyl ester under negative ESI mode. Full scan (c) and MS/MS spectra (d) of indole-3-acetyl-L-methionine methyl ester under positive ESI mode

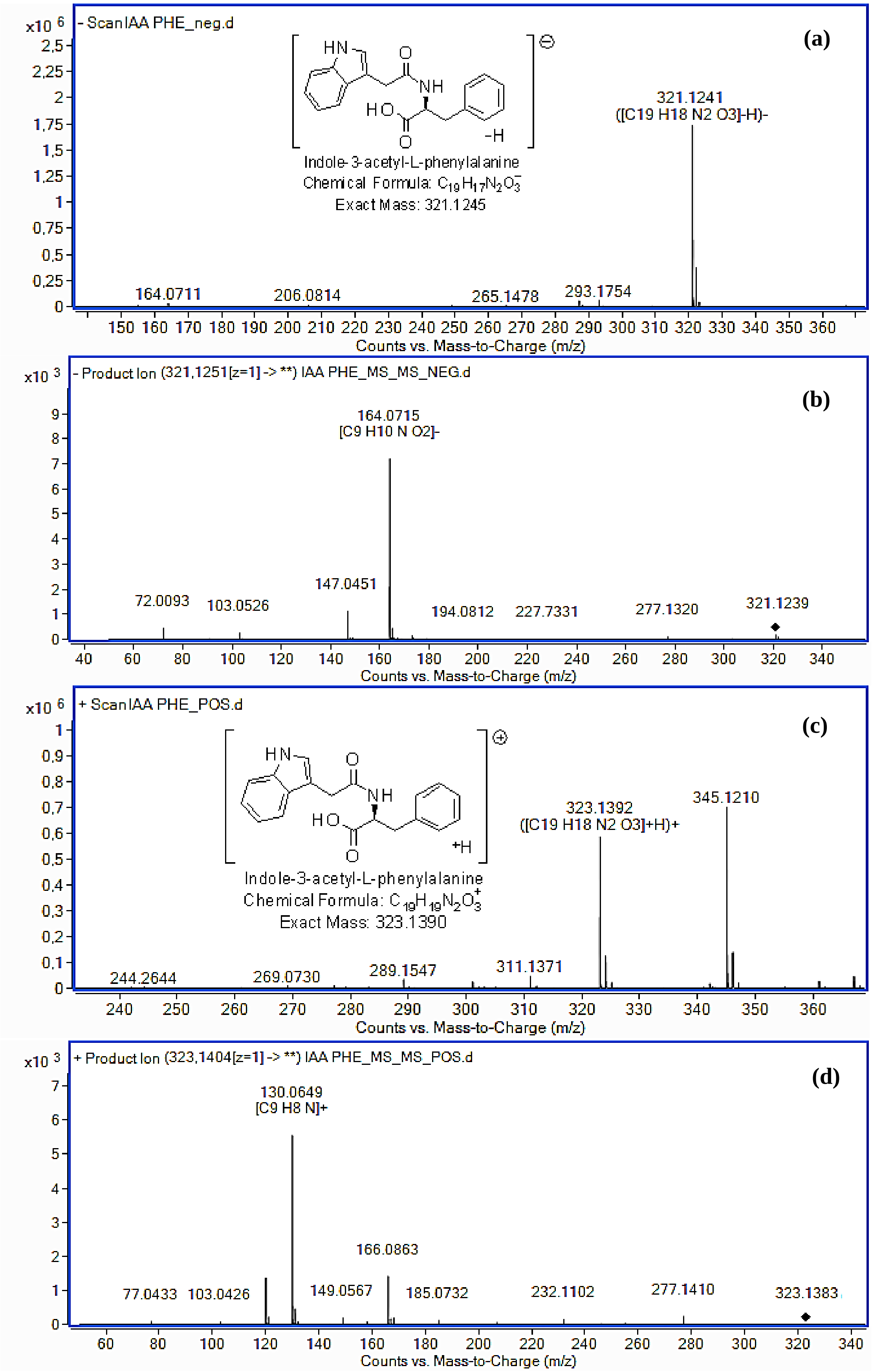

Fig. S14 Full scan (a) and MS/MS spectra (b) of indole-3-acetyl-L-phenylalanine under negative ESI mode. Full scan (c) and MS/MS spectra (d) of indole-3-acetyl-L-phenylalanine under positive ESI mode

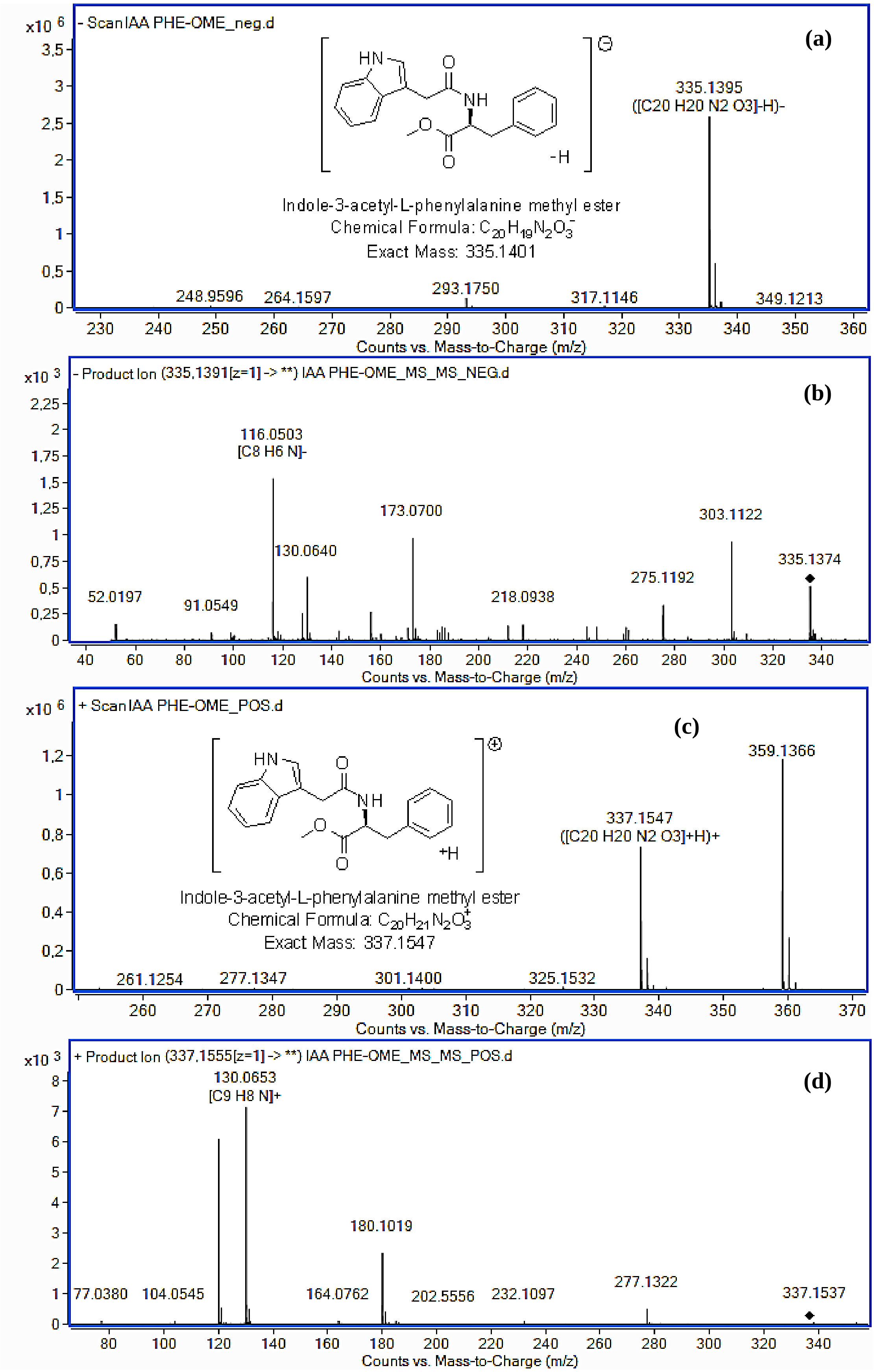

Fig. S15 Full scan (a) and MS/MS spectra (b) of indole-3-acetyl-L-phenylalanine methyl ester under negative ESI mode. Full scan (c) and MS/MS spectra (d) of indole-3-acetyl-L- phenylalanine methyl ester under positive ESI mode

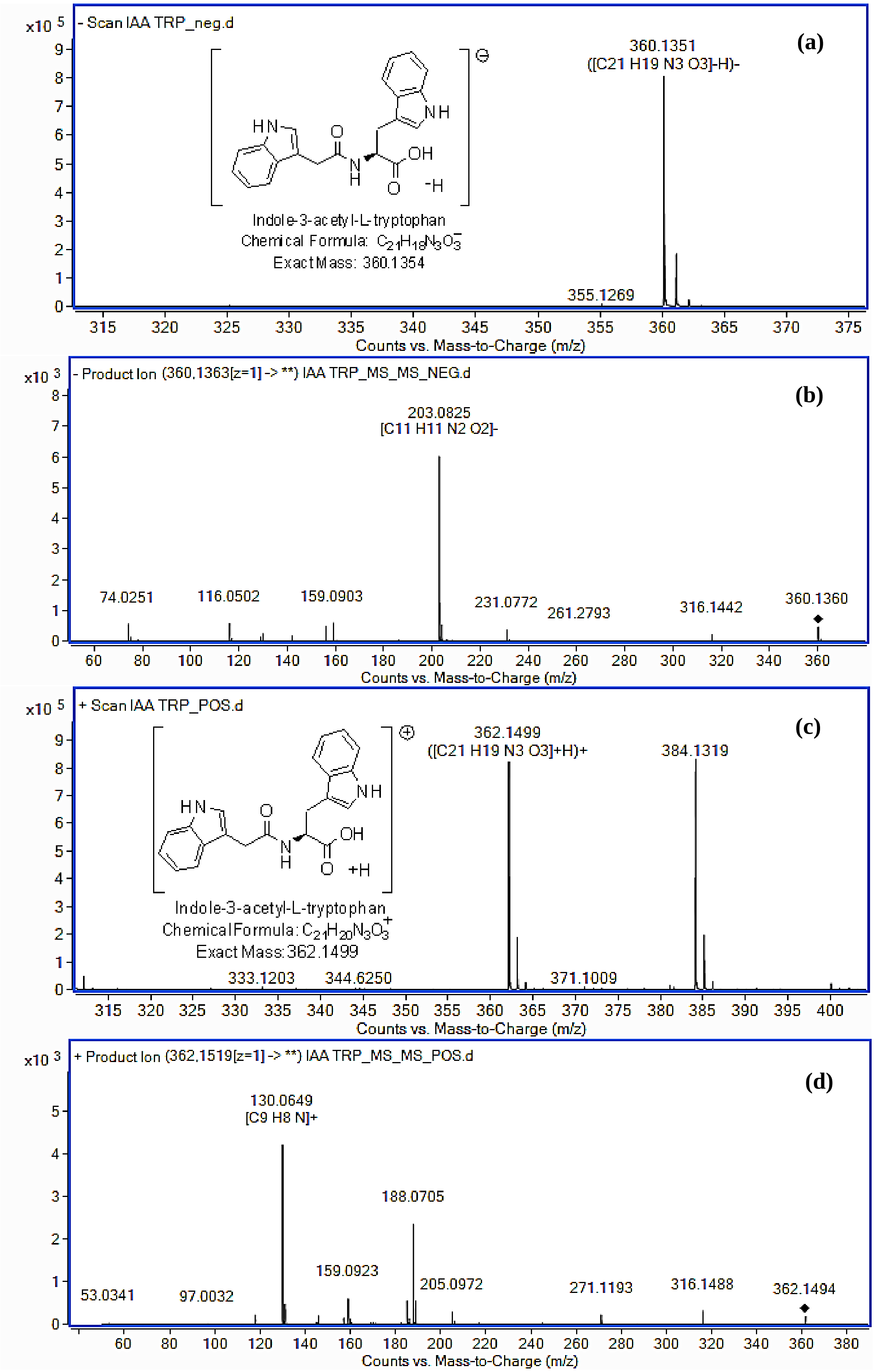

Fig. S16 Full scan (a) and MS/MS spectra (b) of indole-3-acetyl-L-tryptophan under negative ESI mode. Full scan (c) and MS/MS spectra (d) of indole-3-acetyl-L-tryptophan under positive ESI mode

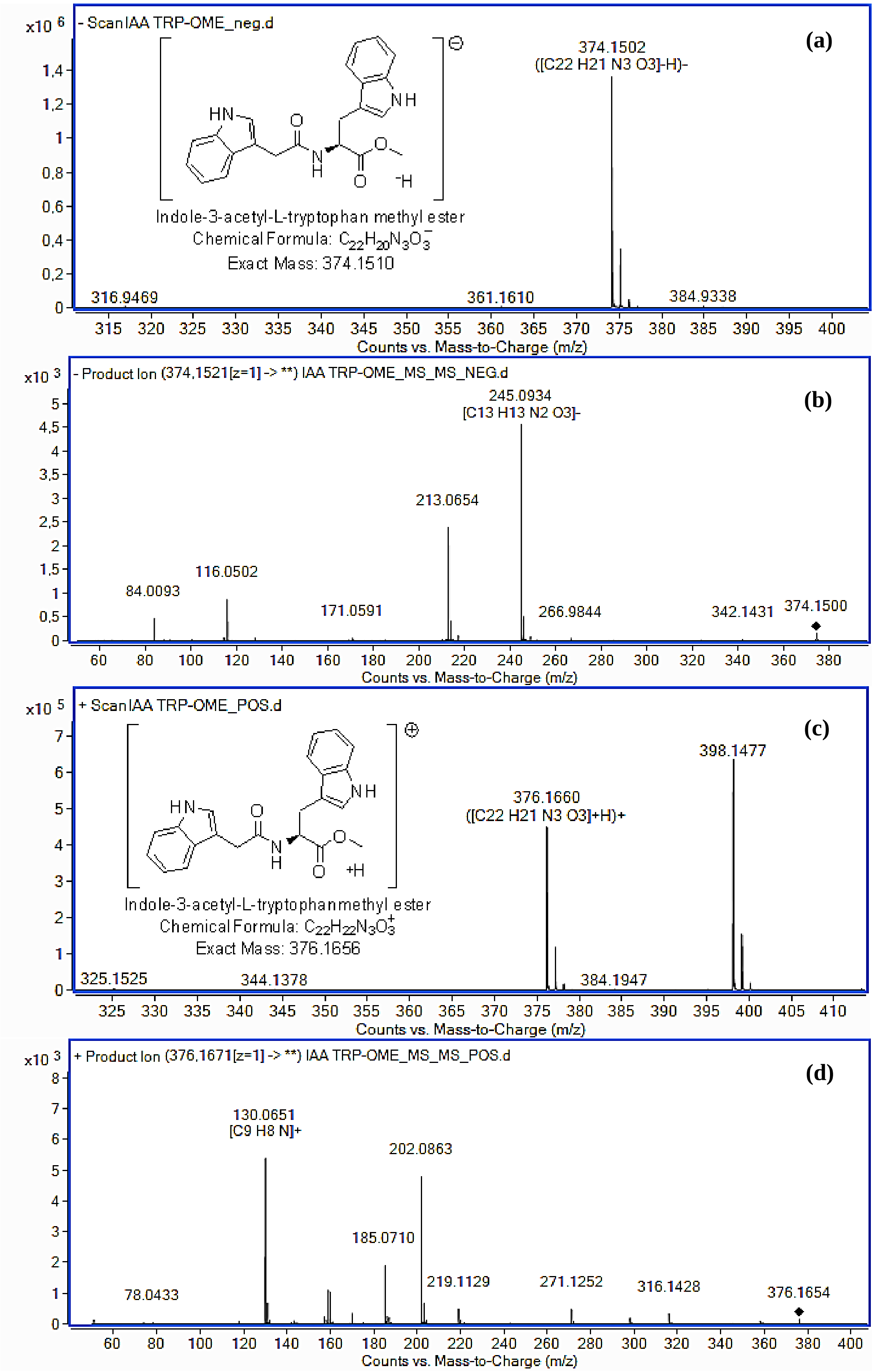

Fig. S17 Full scan (a) and MS/MS spectra (b) of indole-3-acetyl-L-tryptophan methyl ester under negative ESI mode. Full scan (c) and MS/MS spectra (d) of indole-3-acetyl-L-tryptophan methyl ester under positive ESI mode

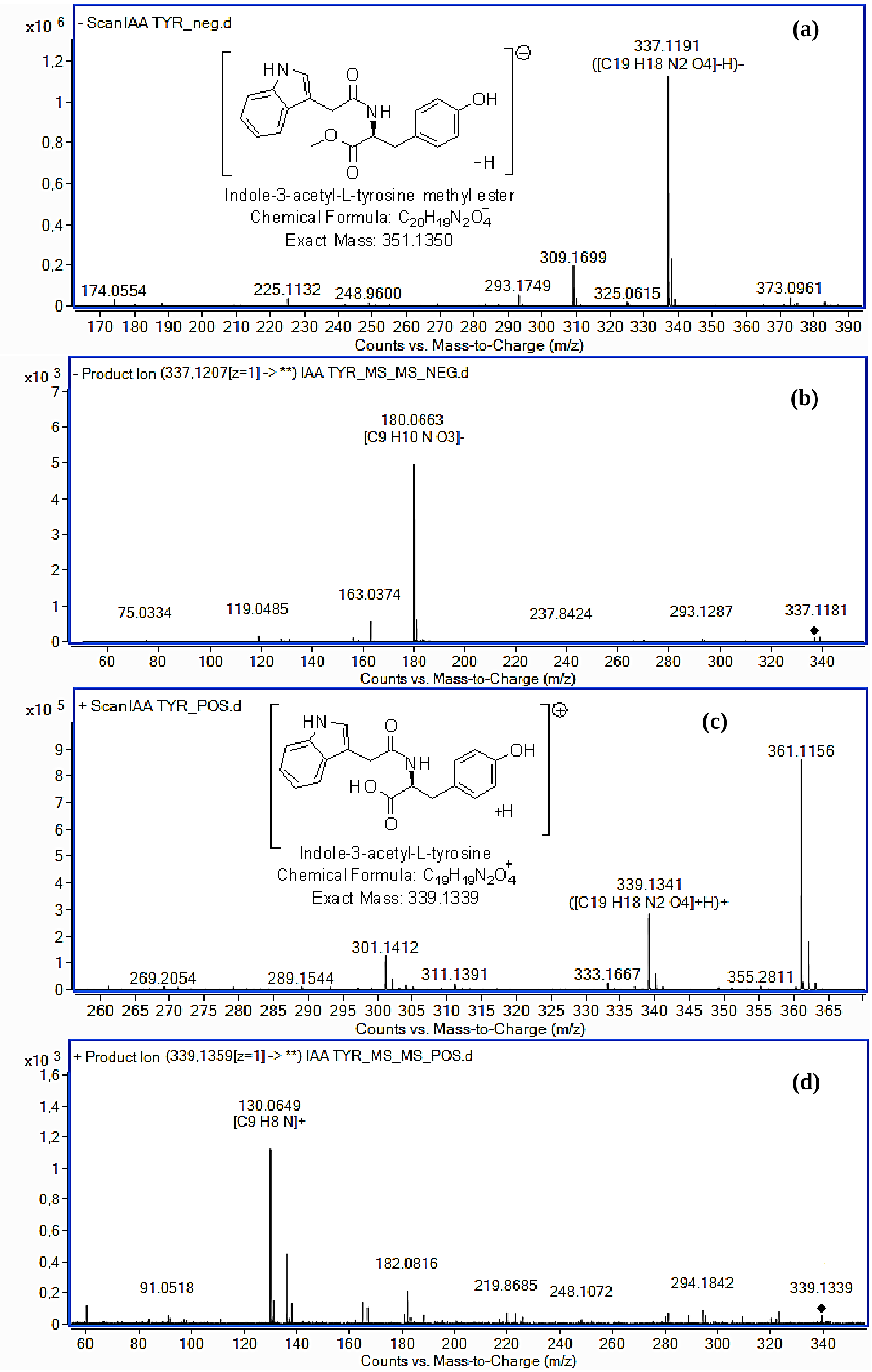

Fig. S18 Full scan (a) and MS/MS spectra (b) of indole-3-acetyl-L-tyrosine under negative ESI mode. Full scan (c) and MS/MS spectra (d) of indole-3-acetyl-L-tyrosine under positive ESI mode

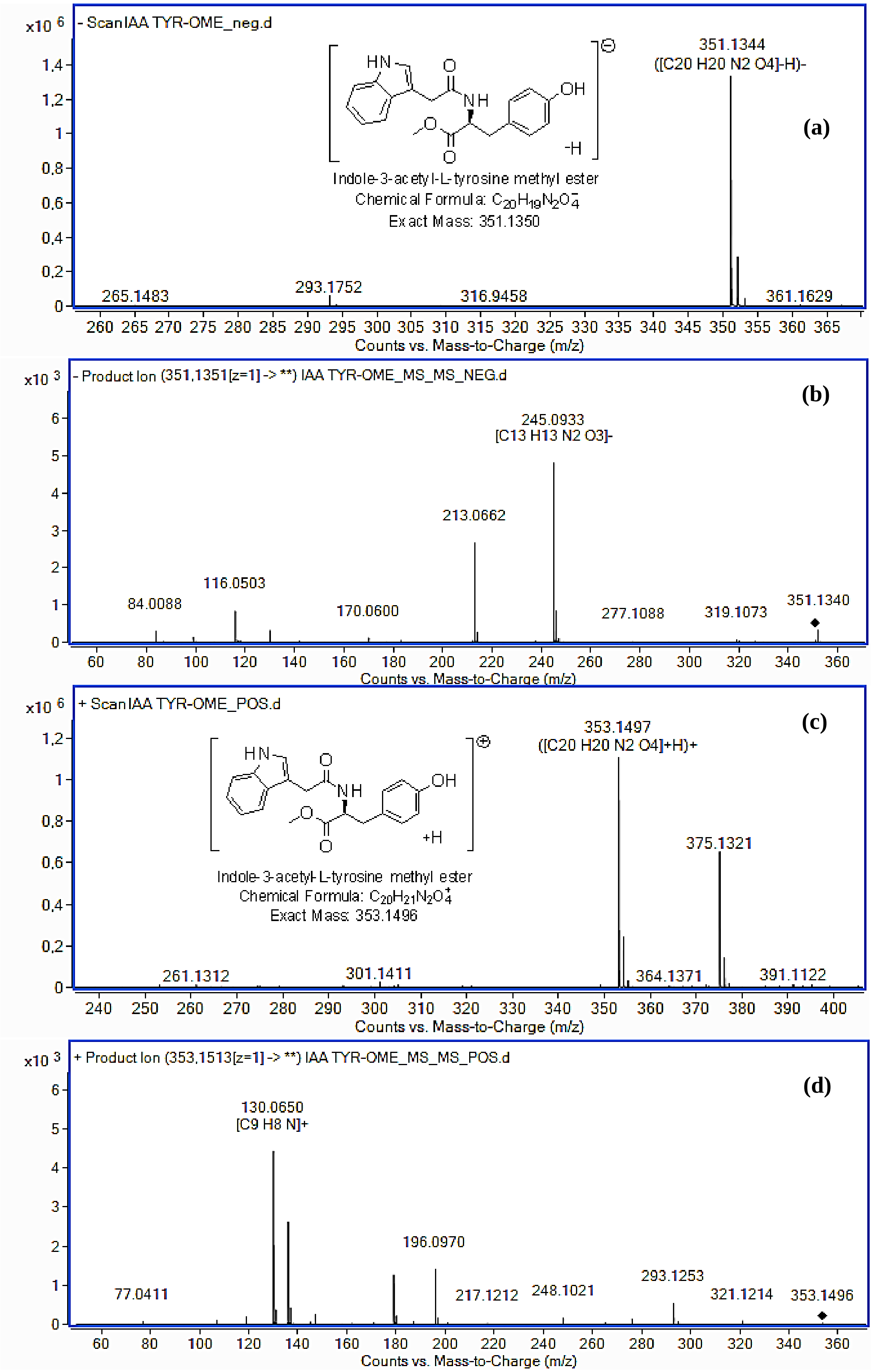

Fig. S19 Full scan (a) and MS/MS spectra (b) of indole-3-acetyl-L-tyrosine methyl ester under negative ESI mode. Full scan (c) and MS/MS spectra (d) of indole-3-acetyl-L-tyrosine methyl ester under positive ESI mode

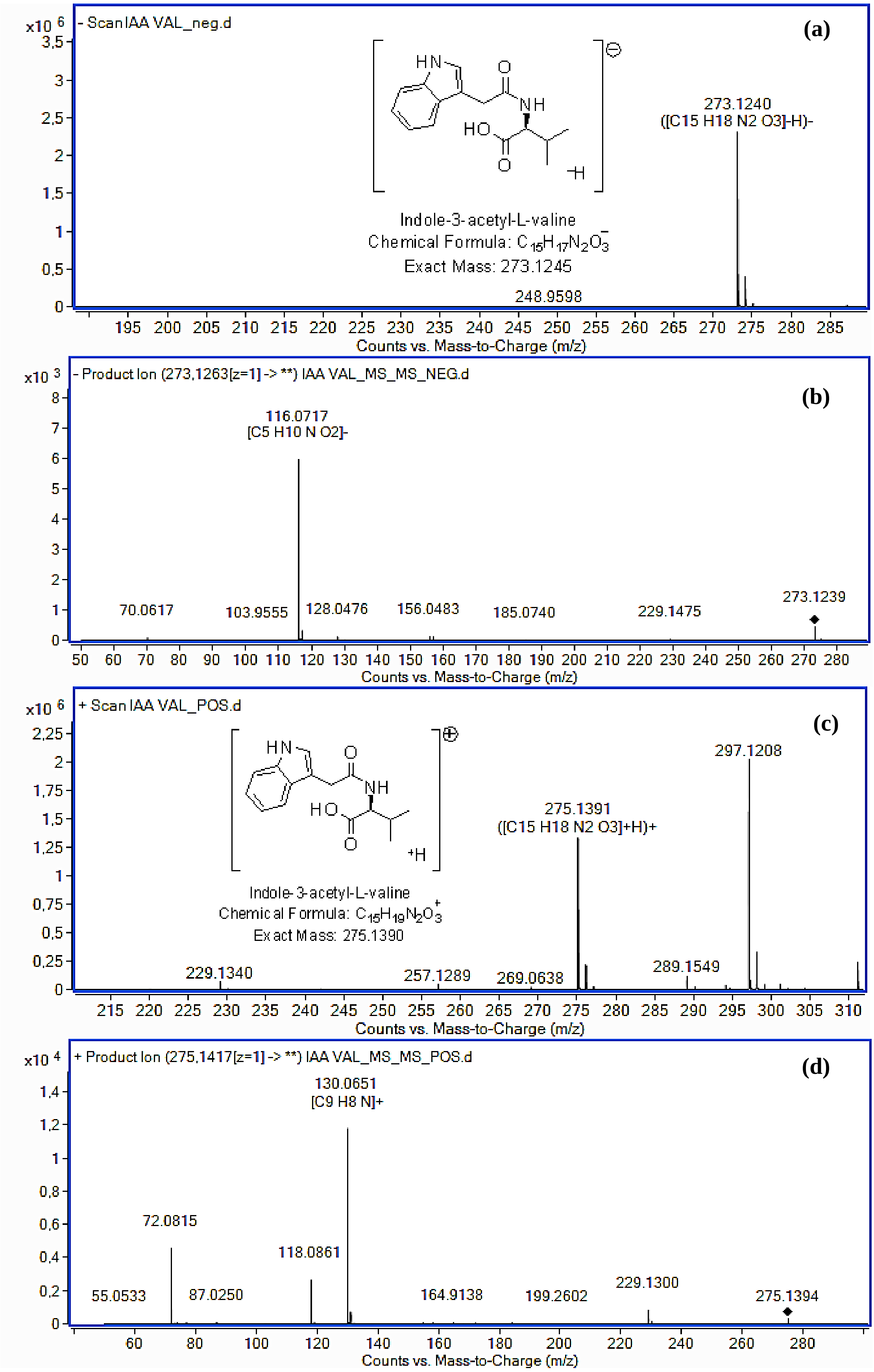

Fig. S20 Full scan (a) and MS/MS spectra (b) of indole-3-acetyl-L-valine under negative ESI mode. Full scan (c) and MS/MS spectra (d) of indole-3-acetyl-L-valine under positive ESI mode

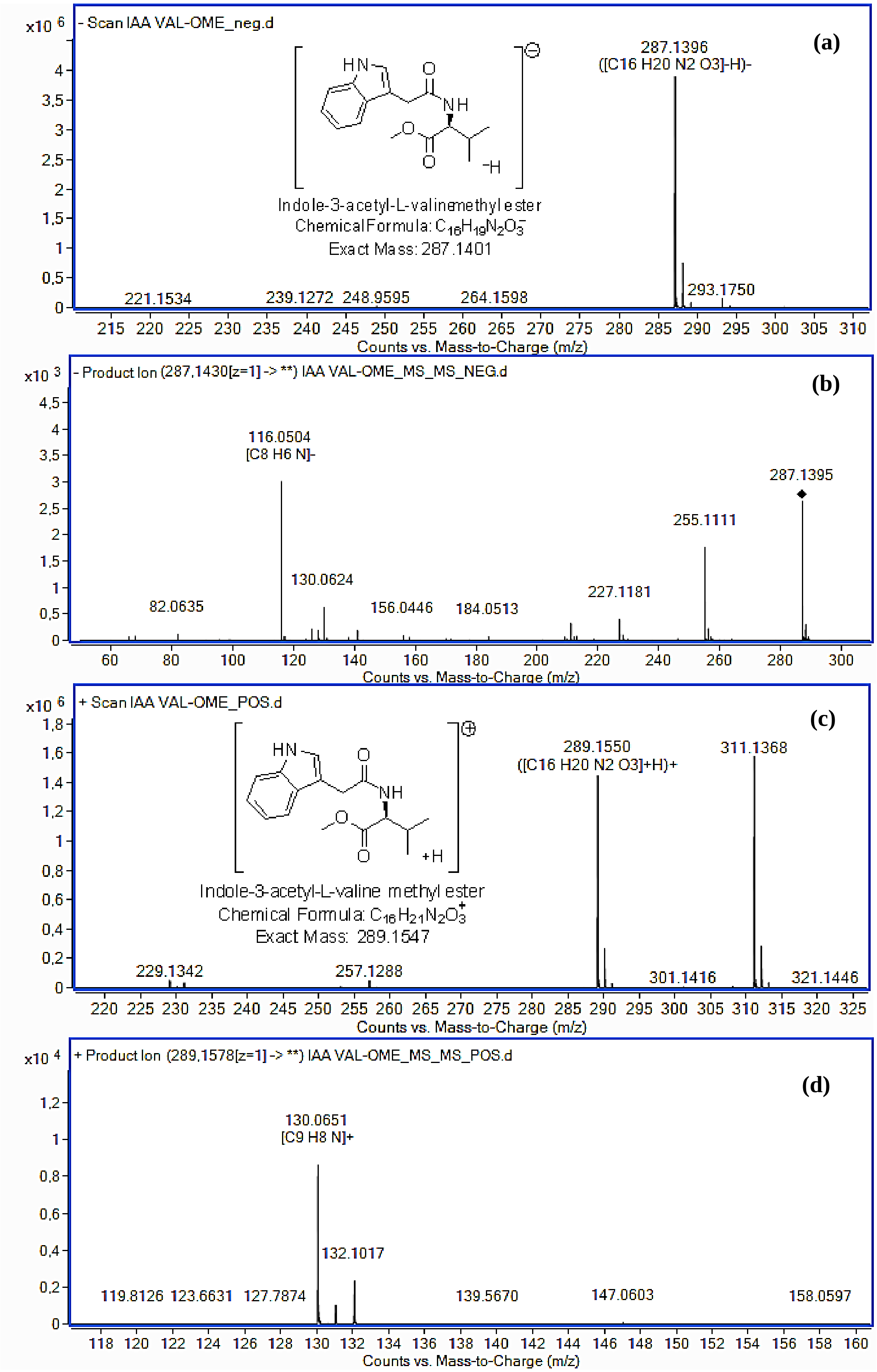

Fig. S21 Full scan (a) and MS/MS spectra (b) of indole-3-acetyl-L-valine methyl ester under negative ESI mode. Full scan (c) and MS/MS spectra (d) of indole-3-acetyl-L-valine methyl ester under positive ESI mode

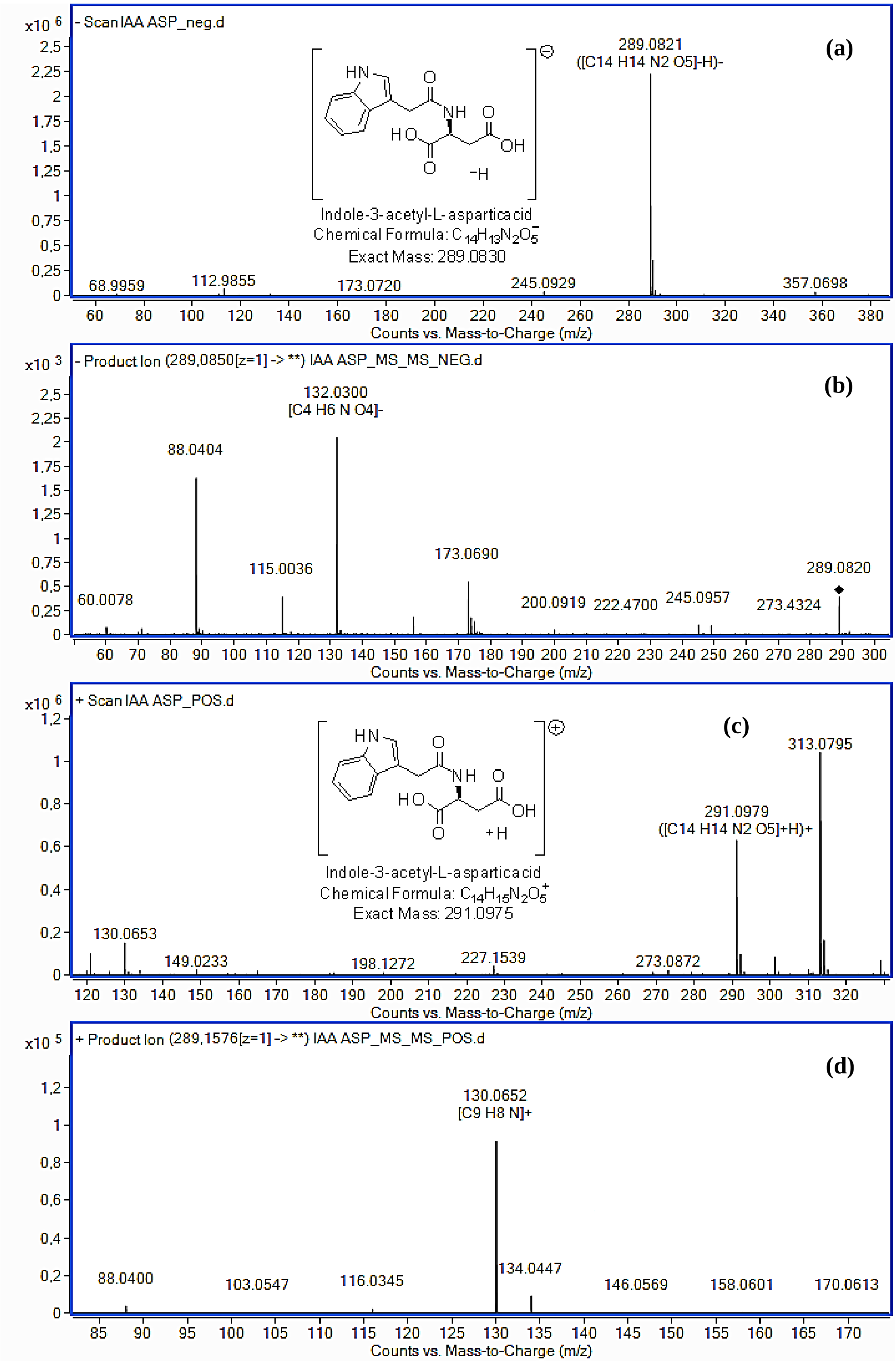

Fig. S22 Full scan (a) and MS/MS spectra (b) of indole-3-acetyl-L-aspartic acid under negative ESI mode. Full scan (c) and MS/MS spectra (d) of indole-3-acetyl-L-aspartic acid under positive ESI mode

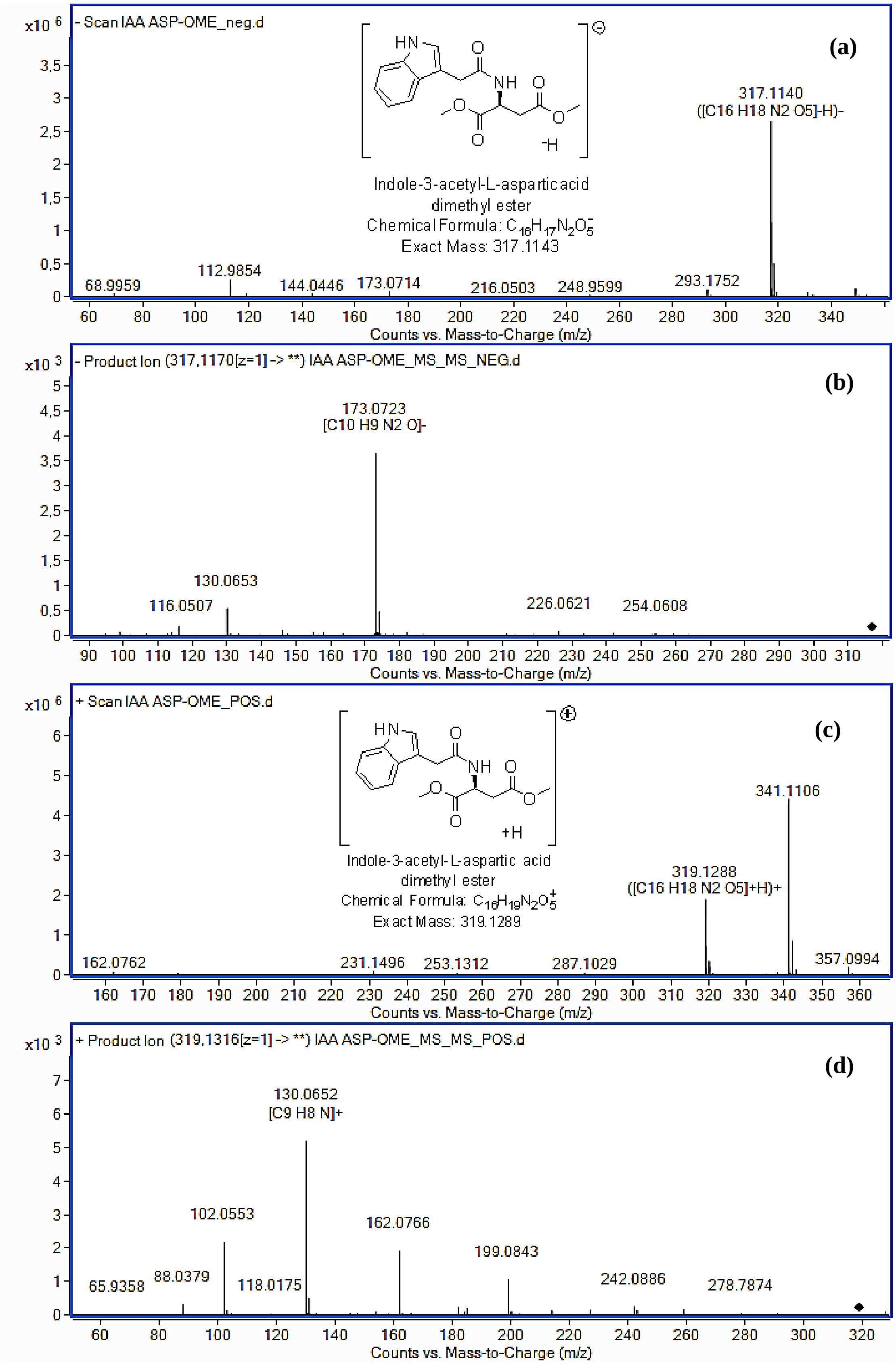

Fig. S23 Full scan (a) and MS/MS spectra (b) of indole-3-acetyl-L-aspartic acid dimethyl ester under negative ESI mode. Full scan (c) and MS/MS spectra (d) of indole-3-acetyl-L-aspartic acid dimethyl ester under positive ESI mode

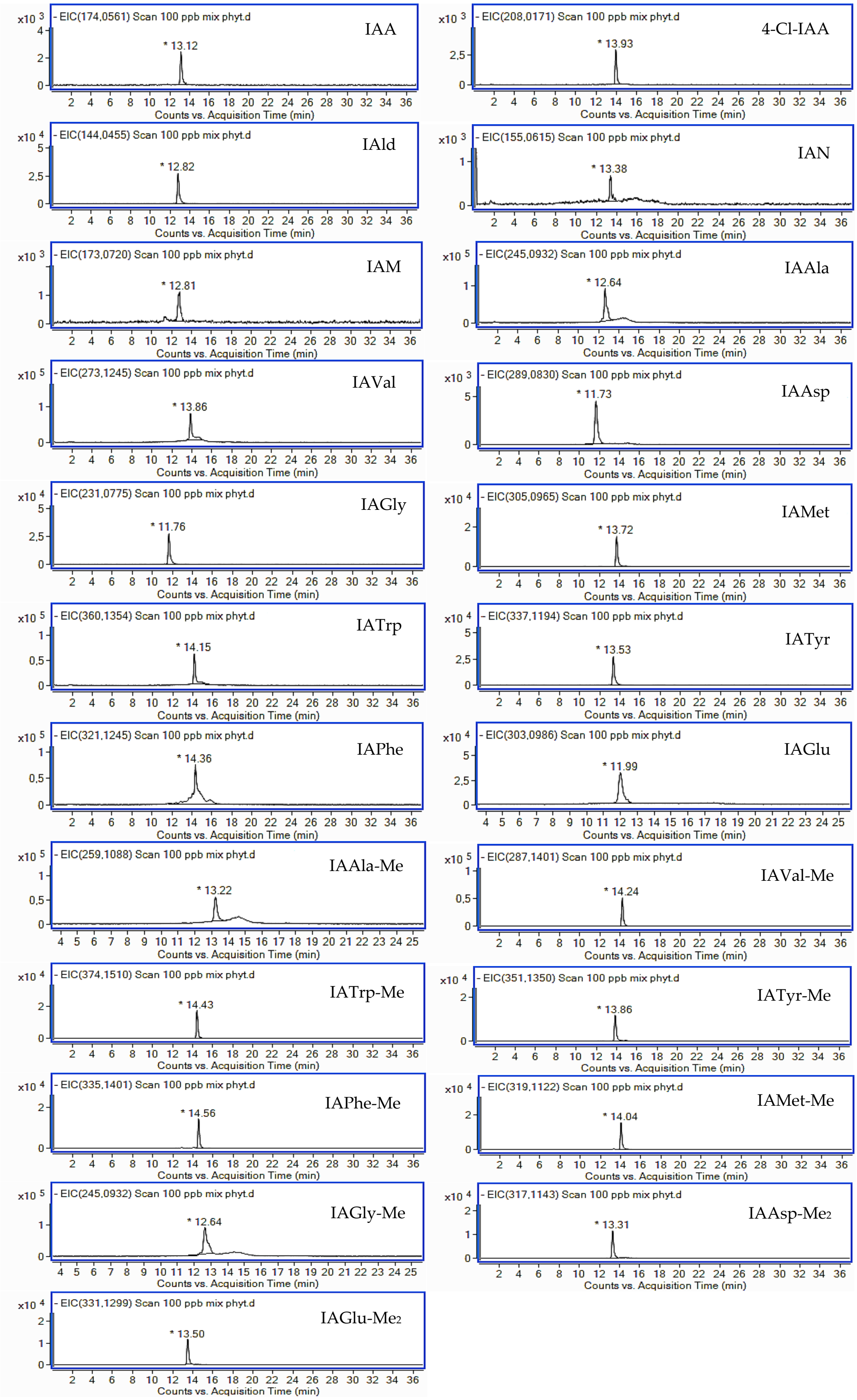

Fig. S24 Extracted ion chromatograms of studied compounds in negative ESI mode

Table S1 MS data of the identification of studied compounds in *B. oleracea* var. *capitata*

Compound name	Elemental composition	Rt[1] (min)	Positive ionization mode $[M+H]^+$				Rt[1] (min)	Negative ionization mode $[M-H]^-$			
			Peak intensity	Calculated mass (*m/z*)	Observed mass (*m/z*)	Mass error		Peak intensity	Calculated mass (*m/z*)	Observed mass (*m/z*)	Mass error
IAA	$C_{10}H_9NO_2$	13.10	$9.1x10^5$	176.0706	176.0706	0	13.12	$1.7x10^5$	174.0561	174.0556	2.9
IAld	$C_9H_7NO_2$	12.82	$1.6x10^6$	146.0600	146.0598	1.4	12.81	$2.5x10^6$	144.0455	144.0451	2.8
IAN	$C_{10}H_8N_2$	13.39	$5.9x10^3$	157.0760	157.0764	2.5	13.38	$9.9x10^3$	155.0615	155.0618	1.9
IAAla	$C_{13}H_{14}N_2O_3$	12.66	$2.3x10^3$	247.1077	247.1069	3.2	12.63	$5.0x10^3$	245.0932	245.0941	3.7
IAVal	$C_{15}H_{18} N_2O_3$	13.87	$5.8x10^4$	275.1390	275.1391	0.4	13.86	$5.9x10^4$	273.1245	273.1245	0
IAGly	$C_{12}H_{12}N_2O_3$	11.76	$3.5x10^2$	233.0921	233.0918	1.3	11.75	$8.2x10^2$	231.0775	231.0771	1.7
IAMet	$C_{15}H_{18}N_2O_3S$	13.70	$1.3x10^3$	307.1111	307.1120	2.9	13.73	$1.7x10^3$	305.0965	305.0975	3.3
IATrp	$C_{21}H_{19}N_3O_3$	14.15	$2.0x10^3$	362.1499	362.1501	0.6	14.17	$2.3x10^3$	360.1354	360.1341	3.6
IAPhe	$C_{19}H_{18}N_2O_3$	14.37	$1.4x10^3$	323.1390	323.1393	0.9	14.35	$2.1x10^3$	321.1245	321.1240	1.6
IAAsp-Me_2	$C_{16}H_{18}N_2O_5$	13.30	$3.0x10^3$	319.1288	319.1280	2.5	13.31	$3.2x10^3$	317.1143	317.1149	1.9
IAPhe-Me	$C_{20}H_{20}N_2O_3$	14.55	$1.0x10^3$	337.1547	337.1550	0.9	14.56	$2.1x10^3$	335.1401	335.1406	1.5
IAGly-Me	$C_{13}H_{14}N_2O_3$	12.66	$2.5x10^3$	247.1077	247.1069	3.2	12.63	$7.0x10^3$	245.0932	245.0935	1.2
IAVal-Me	$C_{16}H_{20}N_2O_3$	14.24	$2.0x10^4$	289.1547	289.1547	0	14.22	$1.8x10^4$	287.1401	287.1406	1.7
IATyr-Me	$C_{20}H_{19}N_2O_4$	13.87	$7.2x10^4$	353.1496	353.1499	0.8	13.86	$7.0x10^4$	351.1350	351.1345	1.4
IASer-Me	$C_{14}H_{16}N_2O_4$	12.34	$6.2x10^4$	277.1183	277.1182	0.4	12.33	$6.8x10^4$	275.1037	275.1032	1.8
IAGlu-Me_2	$C_{17}H_{20}N_2O_5$	13.50	$2.1x10^4$	333.1445	333.1445	0	13.51	$3.0x10^4$	331.1299	331.1295	1.2

[1]Rt: Retention time

Table S2 MS data of the identification of studied compounds in *B. oleracea* var. *rubra*

**Compound name**	**Elemental composition**	**Rt[1] (min)**	**Positive ionization mode $[M+H]^+$**				**Rt[1] (min)**	**Negative ionization mode $[M-H]^-$**			
			**Peak intensity**	**Calculated mass (*m/z*)**	**Observed mass (*m/z*)**	**Mass error**		**Peak intensity**	**Calculated mass (*m/z*)**	**Observed mass (*m/z*)**	**Mass error**
IAA	$C_{10}H_9NO_2$	13.11	$8.8x10^3$	176.0706	176.0709	1.7	13.12	$1.2x10^3$	174.0561	174.0557	2.3
4-Cl-IAA	$C_{10}H_8ClNO_2$	13.90	$2.5x10^2$	210.0316	210.0321	2.4	13.93	$7.3x10^2$	208.0171	208.0165	2.9
			$1.8x10^2$	212.0287	212.0297	4.7		$6.5x10^2$	210.0142	210.0149	3.3
IAld	$C_9H_7NO_2$	12.81	$2.5x10^4$	146.0600	146.0605	3.4	12.80	$4.3x10^4$	144.0455	144.0450	3.5
IAN	$C_{10}H_8N_2$	13.39	$2.9x10^2$	157.0760	157.0755	3.2	13.38	$9.5x10^2$	155.0615	155.0621	3.9
IAAla	$C_{13}H_{14}N_2O_3$	12.67	$6.4x10^2$	247.1077	247.1078	0.4	12.65	$9.1x10^2$	245.0932	245.0944	4.9
IAMet	$C_{15}H_{18}N_2O_3S$	13.72	$5.2x10^3$	307.1111	307.1116	1.6	13.70	$6.0x10^3$	305.0965	305.0972	2.3
IATrp	$C_{21}H_{19}N_3O_3$	14.14	$2.3x10^2$	362.1499	362.1511	3.3	14.12	$3.0x10^2$	360.1354	360.1360	1.7
IAPhe	$C_{19}H_{18}N_2O_3$	14.37	$2.5x10^3$	323.1390	323.1399	2.8	14.35	$3.3x10^3$	321.1245	321.1238	2.2
IAGly-Me	$C_{13}H_{14}N_2O_3$	12.66	$2.0x10^3$	247.1077	247.1088	4.5	12.67	$7.1x10^3$	245.0932	245.0939	2.9
IASer-Me	$C_{14}H_{16}N_2O_4$	12.35	$1.7x10^3$	277.1183	277.1191	2.9	12.33	$1.9x10^3$	275.1037	275.1025	4.4
IAVal-Me	$C_{16}H_{20}N_2O_3$	14.22	$8.1x10^2$	289.1547	289.1545	0.7	14.21	$8.4x10^2$	287.1401	287.1408	2.4

[1]Rt: Retention time

Table S3 MS data of the identification of studied compounds in *B. rapa* subsp. *rapifera*

Compound name	Elemental composition	Rt[1] (min)	Positive ionization mode $[M+H]^+$				Rt[1] (min)	Negative ionization mode $[M-H]^-$			
			Peak intensity	Calculated mass (*m/z*)	Observed mass (*m/z*)	Mass error		Peak intensity	Calculated mass (*m/z*)	Observed mass (*m/z*)	Mass error
IAA	$C_{10}H_9NO_2$	13.13	$8.9x10^4$	176.0706	176.0709	1.7	13.12	$9.6x10^3$	174.0561	174.0556	2.9
IAld	$C_9H_7NO_2$	12.82	$6.9x10^4$	146.0600	146.0603	2.1	12.83	$7.5x10^4$	144.0455	144.0453	1.4
IAN	$C_{10}H_8N_2$	13.37	$2.5x10^2$	157.0760	157.0755	3.2	13.38	$8.0x10^2$	155.0615	155.0615	0
IAAla	$C_{13}H_{14}N_2O_3$	12.64	$2.9x10^3$	247.1077	247.1082	2.0	12.66	$6.6x10^3$	245.0932	245.0930	0.8
IAVal	$C_{15}H_{18} N_2O_3$	13.87	$1.5x10^3$	275.1390	275.1391	0.4	13.88	$1.9x10^3$	273.1245	273.1238	2.6
IATrp	$C_{21}H_{19}N_3O_3$	14.16	$5.3x10^2$	362.1499	362.1516	4.7	14.14	$6.0x10^2$	360.1354	360.1362	2.2
IAPhe	$C_{19}H_{18}N_2O_3$	14.38	$2.0x10^4$	323.1390	323.1397	2.2	14.33	$3.8x10^4$	321.1245	321.1232	4.0
IAAsp	$C_{14}H_{14}N_2O_5$	11.70	$1.1x10^3$	291.0975	291.0969	2.1	11.74	$1.5x10^3$	289.0830	289.0839	3.1
IAGlu	$C_{15}H_{16}N_2O_5$	11.98	$1.8x10^3$	305.1132	305.1126	2.0	11.97	$4.9x10^3$	303.0986	303.0989	1.0
IATyr-Me	$C_{20}H_{19}N_2O_4$	13.88	$1.6x10^3$	353.1496	353.1491	1.4	13.86	$1.2x10^3$	351.1350	351.1340	2.8
IAAla-Me	$C_{14}H_{16}N_2O_3$	13.22	$1.0x10^4$	261.1234	261.1231	1.1	13.21	$3.4x10^4$	259.1088	259.1080	3.1
IAGly-Me	$C_{13}H_{14}N_2O_3$	12.64	$1.1x10^3$	247.1077	247.1088	4.5	12.66	$5.7x10^3$	245.0932	245.0927	2.0
IAVal-Me	$C_{16}H_{20}N_2O_3$	14.23	$3.6x10^3$	289.1547	289.1548	0.3	14.25	$4.7x10^3$	287.1401	287.1406	1.7
IASer-Me	$C_{14}H_{16}N_2O_4$	12.34	$9.4x10^3$	277.1183	277.1184	0.4	12.33	$9.9x10^3$	275.1037	275.1035	0.7

[1]Rt: Retention time

Table S4 MS data of the identification of studied compounds in *B. oleracea* var. *botrytis* cv. zarka

Compound name	Elemental composition	Rt[1] (min)	Positive ionization mode $[M+H]^+$				Rt[1] (min)	Negative ionization mode $[M-H]^-$			
			Peak intensity	Calculated mass (*m/z*)	Observed mass (*m/z*)	Mass error		Peak intensity	Calculated mass (*m/z*)	Observed mass (*m/z*)	Mass error
IAA	$C_{10}H_9NO_2$	13.11	$2.6x10^5$	176.0706	176.0704	1.1	13.11	$3.0x10^4$	174.0561	174.0557	2.3
IAld	$C_9H_7NO_2$	13.95	$3.8x10^5$	146.0600	146.0601	0.7	13.94	$4.7x10^5$	144.0455	144.0450	3.5
IAN	$C_{10}H_8N_2$	13.39	$1.8x10^3$	157.0760	157.0768	5.1	13.36	$6.0x10^2$	155.0615	155.0613	1.3
IAAla	$C_{13}H_{14}N_2O_3$	12.62	$2.5x10^4$	247.1077	247.1087	4.0	12.65	$5.9x10^4$	245.0932	245.0944	4.9
IAVal	$C_{15}H_{18} N_2O_3$	13.89	$3.3x10^5$	275.1390	275.1392	0.7	13.88	$3.5x10^5$	273.1245	273.1236	3.3
IATrp	$C_{21}H_{19}N_3O_3$	14.15	$1.8x10^3$	362.1499	362.1505	1.7	14.11	$1.9x10^3$	360.1354	360.1354	0
IAGly-Me	$C_{13}H_{14}N_2O_3$	12.62	$2.8x10^3$	247.1077	247.1086	3.6	12.65	$7.9x10^3$	245.0932	245.0928	1.6

[1]Rt: Retention time

Table S5 MS data of the identification of studied compounds in *B. oleracea* var. *italica* cv. calabrese

Compound name	Elemental composition	Rt[1] (min)	Positive ionization mode $[M+H]^+$				Rt[1] (min)	Negative ionization mode $[M-H]^-$			
			Peak intensity	Calculated mass (*m/z*)	Observed mass (*m/z*)	Mass error		Peak intensity	Calculated mass (*m/z*)	Observed mass (*m/z*)	Mass error
IAA	$C_{10}H_9NO_2$	13.09	$2.6x10^5$	176.0706	176.0704	1.1	13.11	$3.9x10^4$	174.0561	174.0557	2.3
IAld	$C_9H_7NO_2$	12.81	$5.0x10^5$	146.0600	146.0602	1.4	12.80	$6.7x10^5$	144.0455	144.0452	2.1
IAN	$C_{10}H_8N_2$	13.39	$1.4x10^3$	157.0760	157.0759	0.6	13.36	$6.3x10^3$	155.0615	155.0612	1.9
IAAla	$C_{13}H_{14}N_2O_3$	12.63	$2.1x10^4$	247.1077	247.1078	0.4	12.66	$6.5x10^4$	245.0932	245.0931	0.4
IAVal	$C_{15}H_{18}$ N_2O_3	13.85	$2.0x10^4$	275.1390	275.1392	0.7	13.88	$3.4x10^3$	273.1245	273.1234	4.0
IATrp	$C_{21}H_{19}N_3O_3$	14.14	$5.2x10^3$	362.1499	362.1500	0.3	14.15	$6.0x10^3$	360.1354	360.1345	2.5
IAPhe	$C_{19}H_{18}N_2O_3$	14.36	$6.7x10^2$	323.1390	323.1399	2.8	14.37	$7.7x10^2$	321.1245	321.1241	1.2
IAGlu	$C_{15}H_{16}N_2O_5$	11.98	$5.0x10^3$	305.1132	305.1144	3.9	11.99	$9.4x10^3$	303.0986	303.0985	0.3
IAGly-Me	$C_{13}H_{14}N_2O_3$	12.63	$1.3x10^2$	247.1077	247.1074	1.2	12.66	$5.5x10^2$	245.0932	245.0939	2.9
IAAla-Me	$C_{14}H_{16}N_2O_3$	13.20	$3.0x10^3$	261.1234	261.1233	0.4	13.23	$6.9x10^3$	259.1088	259.1092	1.5
IAVal-Me	$C_{16}H_{20}N_2O_3$	14.24	$2.0x10^3$	289.1547	289.1550	1.0	14.23	$1.0x10^3$	287.1401	287.1397	1.4
IASer-Me	$C_{14}H_{16}N_2O_4$	12.34	$3.2x10^3$	277.1183	277.1183	0	12.36	$3.5x10^3$	275.1037	275.1043	2.2

[1]Rt: Retention time

Table S6 MS data of the identification of studied compounds in *B. oleracea* var. *italica* cv. violleto

Compound name	Elemental composition	Rt[1] (min)	Positive ionization mode $[M+H]^+$				Rt[1] (min)	Negative ionization mode $[M-H]^-$			
			Peak intensity	Calculated mass (*m/z*)	Observed mass (*m/z*)	Mass error		Peak intensity	Calculated mass (*m/z*)	Observed mass (*m/z*)	Mass error
IAA	$C_{10}H_9NO_2$	13.10	$1.1x10^6$	176.0706	176.0700	3.4	13.11	$1.3x10^5$	174.0561	174.0556	2.9
IAld	$C_9H_7NO_2$	12.82	$3.9x10^5$	146.0600	146.0598	1.4	12.80	$4.8x10^5$	144.0455	144.0453	1.4
IAN	$C_{10}H_8N_2$	13.39	$2.0x10^3$	157.0760	157.0764	2.5	13.38	$8.2x10^3$	155.0615	155.0615	0
IAM	$C_{10}H_{10}N_2O$	12.80	$7.4x10^3$	175.0866	175.0870	2.3	12.79	$1.5x10^3$	173.0720	173.0724	2.3
IAAla	$C_{13}H_{14}N_2O_3$	12.67	$4.1x10^3$	247.1077	247.1076	0.4	12.65	$8.8x10^3$	245.0932	245.0937	2.0
IAVal	$C_{15}H_{18} N_2O_3$	13.86	$3.4x10^4$	275.1390	275.1391	0.4	13.86	$3.8x10^4$	273.1245	273.1249	1.5
IATrp	$C_{21}H_{19}N_3O_3$	14.14	$3.2x10^4$	362.1499	362.1514	4.1	14.13	$3.0x10^4$	360.1354	360.1356	0.6
IASer	$C_{13}H_{14}N_2O_4$	11.29	$1.0x10^2$	263.1026	263.1020	2.3	11.28	$1.6x10^2$	261.0881	261.0890	3.4
IAPhe	$C_{19}H_{18}N_2O_3$	14.36	$3.8x10^4$	323.1390	323.1401	3.4	14.38	$4.5x10^4$	321.1245	321.1258	4.0
IAVal-Me	$C_{16}H_{20}N_2O_3$	14.28	$2.0x10^3$	289.1547	289.1545	0.7	14.24	$4.6x10^3$	287.1401	287.1405	1.4
IATyr-Me	$C_{20}H_{19}N_2O_4$	13.89	$1.5x10^3$	353.1496	353.1487	2.5	13.87	$1.1x10^3$	351.1350	351.1341	2.6
IASer-Me	$C_{14}H_{16}N_2O_4$	12.33	$3.3x10^4$	277.1183	277.1187	1.4	12.32	$5.0x10^4$	275.1037	275.1031	2.2

[1]Rt: Retention time

Table S7 MS data of the identification of studied compounds in *R. raphanistrum* subsp. *sativus*

Compound name	Elemental composition	Rt[1] (min)	Positive ionization mode $[M+H]^+$				Rt[1] (min)	Negative ionization mode $[M-H]^-$			
			Peak intensity	Calculated mass (*m*/*z*)	Observed mass (*m*/*z*)	Mass error		Peak intensity	Calculated mass (*m*/*z*)	Observed mass (*m*/*z*)	Mass error
IAA	$C_{10}H_9NO_2$	13.12	$2.3x10^5$	176.0706	175.0705	0.6	13.11	$4.0x10^4$	174.0561	174.0559	1.1
IAld	$C_9H_7NO_2$	12.80	$8.7x10^4$	146.0600	146.0599	0.7	12.79	$9.5x10^4$	144.0455	144.0452	2.1
IAN	$C_{10}H_8N_2$	13.37	$1.7x10^2$	157.0760	157.0767	4.5	13.35	$7.0x10^2$	155.0615	155.0622	4.5
4-Cl-IAA	$C_{10}H_8ClNO_2$	13.91	$1.1x10^2$	210.0316	210.0320	1.9	13.90	$6.0x10^2$	208.0171	208.0178	3.4
			$0.4x10^2$	212.0287	212.0280	3.3		$5.3x10^2$	210.0142	210.0133	4.3
IAM	$C_{10}H_{10}N_2O$	12.81	$8.1x10^2$	175.0866	175.0861	2.9	12.82	$1.4x10^2$	173.0720	173.0724	2.3
IAAla	$C_{13}H_{14}N_2O_3$	12.64	$4.7x10^4$	247.1077	247.1075	0.8	12.65	$8.0x10^4$	245.0932	245.0922	4.1
IAVal	$C_{15}H_{18} N_2O_3$	13.85	$1.4x10^4$	275.1390	275.1388	0.7	13.87	$4.4x10^4$	273.1245	273.1245	0
IAGly	$C_{12}H_{12}N_2O_3$	11.74	$1.6x10^3$	233.0921	233.0928	3.0	11.76	$6.2x10^3$	231.0775	231.0772	1.3
IATrp	$C_{21}H_{19}N_3O_3$	14.16	$1.7x10^3$	362.1499	362.1499	0	14.15	$1.6x10^3$	360.1354	360.1342	3.3
IATyr	$C_{19}H_{18}N_2O_4$	13.54	$1.3x10^3$	339.1339	339.1344	1.5	13.53	$1.4x10^3$	337.1194	337.1197	0.9
IASer	$C_{13}H_{14}N_2O_4$	11.29	$1.6x10^3$	263.1026	263.1029	1.1	11.29	$1.9x10^3$	261.0881	261.0884	1.1
IAPhe	$C_{19}H_{18}N_2O_3$	14.37	$1.9x10^3$	323.1390	323.1392	0.6	14.39	$2.8x10^3$	321.1245	321.1255	3.1
IAGlu	$C_{15}H_{16}N_2O_5$	11.97	$2.7x10^2$	305.1132	305.1137	1.6	11.99	$7.2x10^2$	303.0986	303.0973	4.3
IAAla-Me	$C_{14}H_{16}N_2O_3$	13.20	$3.8x10^3$	261.1234	261.1236	0.8	13.19	$7.5x10^3$	259.1088	259.1090	0.8
IAVal-Me	$C_{16}H_{20}N_2O_3$	14.24	$1.8x10^5$	289.1547	289.1549	0.7	14.24	$3.3x10^5$	287.1401	287.1394	2.4
IAGly-Me	$C_{13}H_{14}N_2O_3$	12.64	$2.8x10^3$	247.1077	247.1089	4.9	12.65	$6.9x10^3$	245.0932	245.0941	3.7
IATyr-Me	$C_{20}H_{19}N_2O_4$	13.86	$1.5x10^3$	353.1496	353.1489	2.0	13.87	$1.8x10^3$	351.1350	351.1342	2.3

IASer-Me	$C_{14}H_{16}N_2O_4$	12.32	2.9x10^3	277.1183	277.1182	0.4	12.32	3.2x10^3	275.1037	275.1044	2.5

[1]Rt: Retention time

Table S8 MS data of the identification of studied compounds in *E. sativa*

Compound name	Elemental composition	Rt[1] (min)	Positive ionization mode $[M+H]^+$				Rt[1] (min)	Negative ionization mode $[M-H]^-$			
			Peak intensity	Calculated mass (*m*/*z*)	Observed mass (*m*/*z*)	Mass error		Peak intensity	Calculated mass (*m*/*z*)	Observed mass (*m*/*z*)	Mass error
IAA	$C_{10}H_9NO_2$	13.13	1.7x10^4	176.0706	176.0709	1.7	13.14	1.2x10^3	174.0561	174.0557	2.3
IAld	$C_9H_7NO_2$	12.83	1.7x10^5	146.0600	146.0596	2.7	12.82	2.1x10^5	144.0455	144.0451	2.8
IAAla	$C_{13}H_{14}N_2O_3$	12.64	5.0x10^3	247.1077	247.1081	1.6	12.67	8.1x10^3	245.0932	245.0934	0.8
IAGly	$C_{12}H_{12}N_2O_3$	11.79	3.2x10^2	233.0921	233.0928	3.0	11.80	8.5x10^2	231.0775	231.0780	2.2
IAVal-Me	$C_{16}H_{20}N_2O_3$	14.21	3.8x10^3	289.1547	289.1554	2.4	14.23	7.2x10^3	287.1401	287.1408	2.4
IATyr-Me	$C_{20}H_{19}N_2O_4$	13.89	7.5x10^3	353.1496	353.1508	3.4	13.86	7.9x10^3	351.1350	351.1356	1.7
IASer-Me	$C_{14}H_{16}N_2O_4$	12.34	1.5x10^3	277.1183	277.1190	2.5	12.32	3.2x10^3	275.1037	275.1031	2.2

[1]Rt: Retention time
